# Acyl-CoA synthetase long-chain family member 4—A novel prognostic marker in cutaneous melanoma

**DOI:** 10.3389/fmed.2025.1553961

**Published:** 2026-03-16

**Authors:** Soumya Paria, Rebecca Lapides, Babak Saravi, Anjali Rajagopal, Alina M. Torrez, Peter Kölblinger, Michael Wang-Evers, Dieter Manstein, Alexander A. Navarini, Peter Lazar, Lajos V. Kemény, Grzegorz Sarek, Kaustubh Adhikari, István B. Németh, Elisabeth Roider

**Affiliations:** 1 School of Mathematics and Statistics, Faculty of Science, Technology, Engineering and Mathematics, The Open University, Milton Keynes, United Kingdom; 2 The Robert Larner, M.D. College of Medicine at the University of Vermont, Burlington, VT, United States; 3 Cutaneous Biology Research Center, Department of Dermatology, Massachusetts General Hospital, Harvard Medical School, Charlestown, MA, United States; 4 Department of Oral, Maxillofacial and Facial Plastic Surgery, Medical Faculty and University Hospital Düsseldorf, Heinrich-Heine-University Düsseldorf, Düsseldorf, Germany; 5 Department of Internal Medicine, University of Connecticut, Farmington, CT, United States; 6 Department of Dermatology, University Hospital of Basel, Basel, Switzerland; 7 Department of Dermatology and Allergology, Paracelsus Medica University, Salzburg, Austria; 8 Department of Oral and Maxillofacial Surgery, Medical Center, University of Szeged, Szeged, Hungary; 9 Department of Dermatology, Venereology and Dermatooncology, Faculty of Medicine, Semmelweis University, Budapest, Hungary; 10 HCEMM-SU Translational Dermatology Research Group, Semmelweis University, Budapest, Hungary; 11 Department of Physiology, Semmelweis University, Budapest, Hungary; 12 Department of Genetics, Evolution and Environment, and UCL Genetics Institute, University College London, London, United Kingdom; 13 Department of Dermatology and Allergology, Szent-Györgyi Albert Medical School, University of Szeged, Szeged, Hungary

**Keywords:** ACSL4, ferroptosis, cutaneous melanoma, survival analysis, immune cell infiltration, prognostic marker, metastatic melanoma

## Abstract

Ferroptosis is an iron-dependent form of regulated cell death driven by lipid peroxidation. Acyl coenzyme A (Acyl-CoA) synthetase long-chain family member 4 (ACSL4) promotes ferroptosis by enriching cellular membranes with polyunsaturated fatty acids, yet its prognostic relevance in melanoma remains unclear. We conducted a retrospective analysis of 63 patients with melanoma to evaluate associations between ACSL4 expression and overall survival (OS), metastasis-free survival (MFS), and disease-free survival (DFS). Correlation analyses and Cox proportional hazards (CoxPH) models were used to identify prognostic factors, and immune cell infiltration was assessed in tumor samples. Higher ACSL4 expression was consistently associated with improved OS, MFS, and DFS in cutaneous melanoma. ACSL4 levels also correlated with greater immune cell infiltration within the tumor microenvironment. Notably, metastatic melanoma cases exhibited higher ACSL4 expression than non-metastatic cases; however, within cutaneous melanoma, elevated ACSL4 remained linked to more favorable survival outcomes. Together, these findings position ACSL4 as a promising prognostic biomarker in melanoma and suggest a potential connection between ferroptosis biology and antitumor immunity. While the study is retrospective and correlative, it provides a strong rationale for prospective validation and mechanistic studies to test whether modulating ACSL4-driven ferroptosis can improve clinical outcomes.

## 1 Introduction

Cells ultimately die through one of three pathways: apoptosis, necrosis, or ferroptosis (1). Apoptosis is a programmed, physiological process in which cells and nuclei shrink and are efficiently cleared by phagocytes. Necrosis is unprogrammed and disorganized; external insults cause swelling, membrane rupture, and premature death (2). Ferroptosis is an iron-dependent process driven by reactive oxygen species generated from oxidized lipids. Unlike apoptosis or necrosis, mitochondria shrink while the plasma membrane, nuclear size, and chromatin architecture remain intact (3).

Numerous regulators influence ferroptosis, including acyl coenzyme A (acyl-CoA) synthetase long-chain family member 4 (ACSL4). This enzyme belongs to a family of five, located on the endoplasmic reticulum and outer mitochondrial membranes, and facilitates the conversion of fatty acids into acyl-CoA esters. These acyl-CoA esters play key roles in fatty acid metabolism, contributing to the modification of cellular membranes (4). Research shows phosphatidylethanolamine (PE) is the major phospholipid in cell membranes that can initiate ferroptosis. ACSL4 and lysophosphatidylcholine acetyltransferase 3 (LPCAT3) are both involved in PE biosynthesis (5). Consequently, lowering ACSL4 or LPCAT3 levels reduces PE, thereby diminishing the cell's capacity to undergo ferroptosis (5).

Previous studies have investigated the impact of ACSL4 on cell sensitivity to ferroptosis. Using a genome-wide CRISPR screen and microarray analysis of ferroptosis-resistant cells, ACSL4 was found to enhance sensitivity to ferroptosis (a new form of cell death), thereby increasing the likelihood of ferroptosis (6). Additional studies support that higher ACSL4 levels increase cell vulnerability to ferroptosis (4, 7). Based on this evidence, ACSL4 is now considered a potential target for treating diseases associated with ferroptosis.

The expression levels of ACSL4 have been studied across different cancers; however, the results remain elusive. A recent review highlights that ACSL4 inhibits the progression of lung cancer (8), estrogen receptor-positive breast cancer (9), and cervical cancer (10, 11), likely due to its ability to increase ferroptosis-mediated cell death through the generation of reactive oxygen species (6). However, evidence indicates that ACSL4 is also highly expressed in other aggressive cancers, such as estrogen receptor-negative breast cancer, hepatocellular carcinoma, colorectal cancer, and prostate cancer, which are characterized by rapid growth, migration, and invasion (6). The data also indicate that elevated ACSL4 levels in cancers that frequently express it are closely linked to a worse prognosis, likely because ACSL4 facilitates uncontrolled cell proliferation, tumor invasion, and resistance to programmed cell death (12). Therefore, it has been proposed that adjusting ACSL4 expression levels to alter the typical pattern in a particular cancer type could offer a promising therapeutic strategy (6).

Melanoma, a form of skin cancer, can be fatal if not detected and treated promptly. Its global incidence has steadily risen over the past 50 years, likely influenced by various contributing factors (13, 14). Understanding the role of ACSL4 expression in melanoma is crucial, as it can deepen understanding of how ACSL4 affects melanoma cells and may inform the development of new therapies and prognostic tools.

This study aimed to investigate the impact of ACSL4 expression on prognostic factors in melanoma, including overall survival (OS), disease-free survival (DFS), metastasis-free survival (MFS), and immune cell infiltration in melanoma cells. The goal was to assess whether ACSL4 expression level can categorize patient risk and correspond with immune infiltration patterns, thus clarifying its potential as a predictor of outcomes in melanoma.

## 2 Methods

### 2.1 Data

The dataset comprises records of 63 patients treated at the Department of Dermatology and Allergology at the University of Szeged. Due to the nature of this retrospective, non-interventional study, informed consent was not obtained from patients, as the patient data were all anonymized before access. The data were accessed within the time frame of 1 January 2000, through 30 June 2023. The melanoma patients were retrospectively selected in the period 1998–2019. Their primary melanomas and metastases were obtained from the database of archived or previously collected tissue blocks during the investigation period. The study and data collection were conducted in accordance with the approved protocols of Mel-Biochip-001 and Mel-Retro-001 (Szeged). The dataset included 12 explanatory variables and four response variables, which will be discussed in detail Section 2.2. A full variable dictionary and coding/transformations are explained in Sections 2.2–2.4 and Supplementary materials S1, S2; approaches used to accommodate repeated ACSL4 measures and clinical heterogeneity [random-effects survival, Akaike Information Criterion (AIC)-guided model selection, Benjamini–Krieger–Yekutieli (BKY) to control the study-wise false discovery rate (FDR)] are detailed in Sections 2.6.3, 2.7, and 2.9; external validation is reported in Sections 2.8–2.11.

The number of ACSL4 observations per patient varied throughout the dataset. Each observation represented one measurement from different areas and melanoma subclones of the same tumor, reflecting the sample's expression pattern. Optical densitometric measurements were derived from the colorimetric reactions of ACSL4 labeling. For the first 20 patients, only three measurements were taken, while five measurements were recorded for the remaining 43 patients. The dataset also included an extra variable tracking ACSL4 observations post-metastasis, but these data were only available for the first 20 patients.

### 2.2 Explanatory variables

The 12 explanatory variables in the dataset can be broadly categorized into three groups:

1. Basic covariates: this category consisted of two variables—the age at melanoma diagnosis and the patient's sex.
2. Genetic mutation: this category included only one variable, BRAF status, which reflected whether a BRAF mutation was present in the patient. This mutation is known to lead to more aggressive growth of melanoma (15).
3. Melanoma characteristics: this set consisted of nine variables that detailed the melanoma features according to histopathology. Supplementary Table S1 provides a comprehensive description of these variables.

### 2.3 Response variables

The four response variables are described below.

1. Status indicates whether the patient is alive or deceased.
2. DFS, or disease-free survival, refers to the period from the initial diagnosis of primary melanoma until the detection of any recurrence or further disease.
3. MFS or metastasis-free survival refers to the duration from the initial diagnosis of primary melanoma to the first instance of metastasis, measured in months.
4. OS: overall survival duration (in months), measured from the melanoma diagnosis date to the last observed date when the patient is still alive.

### 2.4 Variable transformation

The explanatory variables in the dataset included some categorical variables with more than two distinct values, which were unsuitable for direct use in the model. To address this, indicator variables were created for each distinct value. For example, the variable “Type” takes values 0, 1, and 2 depending on the melanoma subtype. 0 corresponds to superficial spreading melanoma (SSM), 1 to nodular melanoma (NM), and 2 to acral lentiginous melanoma (ALM). We created three indicator variables Type0, Type1, and Type2. The indicator variable Type0 takes the value 1 for SSM and 0, otherwise. The indicator variable Type1 takes the value 1 for NM and 0 otherwise. The indicator variable Type2 takes the value 1 in case of ALM and 0 otherwise.

The variable “Loc_m” indicates the various sites where metastasis had occurred in a patient. Since a patient may have multiple metastasis sites, we transformed this variable into a count variable named “Loc_m_cnt.” This count variable represents the total number of distinct metastasis sites for each patient. For instance, if a person has metastases at five different sites, regardless of which specific sites they are, “Loc_m_cnt” would be five. The final list of explanatory variables is provided in Supplementary Table S2.

### 2.5 Significance test of difference in expression

One of our studies aims was to determine if there are significant differences in ACSL4 levels between primary and metastatic tumors. To this end, we divided our data into two groups: patients with primary melanoma only, and those with diagnosed metastasis after primary melanoma. For the first 20 patients with ACSL4 levels available in both primary and metastatic lesions, we used the non-parametric one-sided Wilcoxon signed-rank test to test whether ACSL4 levels are significantly higher in metastatic tumors compared to primary melanoma in each patient.

### 2.6 Survival analysis setup

A survival analysis setup comprises two essential components: the hazard and the survival probability. The probability of surviving until time *t*, called the survival function, is represented as *S*(*t*). The hazard function quantifies the instantaneous rate of event occurrence, such as death, failure, or relapse, given that the event has not occurred up to time *t*.

Mathematically, the hazard function (16) can be expressed as


$$\begin{array}{l} \lambda \left(t\right)=-\frac{d}{dt}\log S\left[t\right]\; \end{array}$$

#### 2.6.1 Kaplan–Meier survival estimate

The Kaplan–Meier Survival Estimate provides a non-parametric estimate of the survival function based on the data. At the starting point of the study, *t*_0_ = 0, all the patients are alive, so *S*(*t*_0_) = 1. Then the time points of each event are ordered, with *t*_1_ being the time of the first event, etc. The survival probability at time *t*_*i*_ can be estimated as (17):


$$\begin{array}{l} S\left(t_{i}\right)=S\left(t_{i-1}\right)\left(1-\frac{d_{i}}{n_{i}}\right)\; \end{array}$$

where *n*_*i*_ is the number of patients alive just before *t*_*i*_, and *d*_*i*_ is the number of events at *t*_*i*_. This formulation allows for the possibility of multiple events occurring simultaneously.

The Kaplan–Meier curve is the plot of this estimated survival probability against time. Typically, the population is divided into groups based on covariate levels, and Kaplan–Meier curves for each group are plotted together for comparison. The survival curves between two groups can be compared using the log–rank test. This is a non-parametric test that compares the estimates of hazard functions between the two groups under the null hypothesis that the hazard functions of the two groups are identical.

We employed Kaplan–Meier curves and log–rank tests to compare different covariate levels in our dataset. For continuous variables such as age, we split the data into two groups based on the median value of the covariate and then compared these groups.

#### 2.6.2 Cox proportional hazards (CoxPH) model

The proportional hazards model assumes that the hazard rates of different individuals remain in the same proportion at all time points. Hence, the hazard function at time *t* for a patient *i* with covariates *z*_*i*_ can be expressed as the product of baseline hazard, λ_0_(*t*), and *g*(*z*_*i*_), a function of the covariates (16):


$$\begin{array}{l} \lambda \left(t;z_{i}\right)=\lambda_{0}\left(t\right)\;g\left(z_{i}\right)\; \end{array}$$

In this equation, *g*(*z*_*i*_) is a function solely determined by the covariates for the patient *i* and is independent of time *t*.

Additionally, the Cox model assumes that the natural logarithm of *g*(*z*_*i*_) can be represented as a linear function of the covariates (16):


$$\begin{array}{l} \log g\left(z_{i}\right)=\beta_{0}+\beta_{1}z_{i1}+\beta_{2}z_{i2}+\ldots +\beta_{k}z_{ik}\; \end{array}$$

This leads to the following formulation for the hazard function:


$$\begin{array}{l} \lambda \left(t;z_{i}\right)=\lambda_{0}\left(t\right)\;\exp\left(\beta_{0}+\beta_{1}z_{i1}+\beta_{2}z_{i2}+\ldots +\beta_{k}z_{ik}\right)\; \end{array}$$

The vector of linear coefficients, denoted as β, can be estimated by maximizing the partial likelihood function.

#### 2.6.3 Model selection

The final multivariate models for each response variable were selected from a set of candidate models based on the Akaike Information Criterion (AIC). AIC is a measure that compares the quality of different models based on their relative fit given a set of candidate models. The AIC value for a model is calculated as $AIC=2k-2\ln\left(\hat{L}\right)$, where *k* is the number of model parameters and $\hat{L}$ is the maximum likelihood value for the model. A lower AIC value indicates a better model fit, as it balances the trade-off between model complexity (in terms of the number of parameters) and goodness of fit. A model with a lower AIC value is preferred over others in the set of candidate models, as it offers a better fit while using fewer parameters.

### 2.7 Data preparation

The dataset exhibited non-uniformity regarding the number of ACSL4 observations per patient. Each observation of ACSL4 corresponded to one measurement from the same tumor, but from a randomly different area. For the first 20 patients, only three measurements were taken, but five measurements were taken for the remaining 43 patients.

To address this, instead of summarizing the ACSL4 values for each patient, we opted to consider each ACSL4 value as a separate observation. Consequently, for each patient, we had multiple rows with ACSL4 values, while the other variables remained constant. This revised dataset was utilized for fitting survival models. In recognition of the fact that each patient has multiple observations, we used a random effects model within the survival setup, with the patient's identity (ID) as a dummy variable.

### 2.8 External validation: TCGA

It is important to note that the variable with the highest number of complete observations is DFS, whereas OS and MFS have a substantial number of right-censored observations. The right censoring means that for OS and MFS, there were individuals whose exact duration of survival was not known because they were still alive at the end of the follow-up period, which extended until 2020. As the study could not continue to track them beyond that point, the survival time for these individuals is considered censored. Consequently, the effective number of observations was smaller for these two variables. Furthermore, the dataset itself is relatively small, so to validate and replicate our findings, a larger cohort was required. To address this, we utilized publicly available The Cancer Genome Atlas (TCGA) data to reproduce our observations. This external validation was explicitly planned to mitigate concerns about generalizability arising from a single-center discovery cohort (see also Sections 2.10 and 2.11).

The TCGA data was considerably larger than our dataset, comprising 480 records. Out of these samples, 371 were derived from patients with metastatic melanoma, while the remaining 109 were from patients with primary melanoma. Our first objective was to assess whether there were statistically significant differences in ACSL4 levels between the two groups. As the set of patients was different for primary and metastatic melanoma measurements, we could not employ a pairwise comparison similar to our main cohort; we thus employed a linear regression to assess the differences in ACSL4 levels between the two groups, adjusted for the covariates age and sex. Then, for each of the two tumor types (primary and metastatic), we fitted multivariate CoxPH models (with age, sex, and ACSL4) to the TCGA data to assess whether ACSL4 is a significant predictor of survival. In our initial data, we were unable to fit a separate model for metastatic tumors due to the small sample size. We also examined the Kaplan–Meier curve to assess the direction and nature of the association between ACSL4 and survival in the TCGA data.

### 2.9 Adjustment for multiple testing

After performing numerous significance tests for the survival analysis results, we adjusted for multiple comparisons using the Benjamini–Krieger–Yekutieli (BKY) method (18) to control the study-wise false discovery rate (FDR). The significance threshold determined by the FDR method was 0.0099 based on the presented *p*-values. The *p*-values at or below this threshold were deemed significant, while those below the unadjusted threshold of 0.05 were only considered nominally significant.

### 2.10 External validation: TIDE

Apart from validating our findings in the TCGA dataset, we sought additional confirmation through Kaplan–Meier curves provided on the Tumor Immune Dysfunction and Exclusion (TIDE) website (19). The TIDE website hosts Kaplan–Meier curves from various previous survival analysis studies. These curves illustrate the survival rates of patients divided into two groups based on the median ACSL4 value in the data. We examined these curves to determine whether our observations on ACSL4 align with those reported in previous studies.

### 2.11 External validation: TIMER

Finally, we explored the relationship between ACSL4 expression and the invasion of tumor immune-infiltrating cells (TIIC) using the Tumor Immune Estimation Resource (TIMER) (20). TIMER is a web platform designed to systematically assess the clinical impact of various immune cells across different types of cancer. Using this tool, we created scatter plots to illustrate the relationship between TIIC estimates and ACSL4 expression. Since most immune cell types are negatively correlated with tumor purity, and tumor purity is a significant confounding factor, we used TIIC estimates that were adjusted for purity.

#### 2.11.1 Immune infiltration estimation and ACSL4-TIIC association analysis

We quantified tumor-infiltrating immune cells (TIICs) using the TIMER platform (version 2.0; (20)) for the TCGA Skin Cutaneous Melanoma (SKCM) dataset. For each TCGA-SKCM sample, TIMER provides estimated abundances for six canonical lineages (B cells, CD4^4^ T cells, CD8^4^ T cells, neutrophils, macrophages, dendritic cells) derived from bulk RNA-seq through constrained regression on lineage-specific marker sets. To assess the relationship between ACSL4 expression and immune infiltration, we utilized TIMER's gene-immune correlation workflow with tumor-purity adjustment enabled (partial Spearman correlation), retrieving correlation coefficients and *p*-values for each lineage. Where applicable, we inspected additional microenvironment annotations available in TIMER (e.g., CAFs/monocytes) to contextualize trends shown in Supplementary Figure S6. All *p*-values from these analyses were included in the study-wide BKY FDR procedure reported in Section 2.9; significant associations are those with FDR ≤ 0.0099, and nominal associations are reported for completeness.

### 2.12 Tissue microarray (TMA), immunohistochemistry (IHC), visualization, and detection methods

Tissue microarrays (TMAs) were prepared from paraffin-embedded tissue blocks of both primary tumors and metastases from the 63 melanoma patients described earlier. From the original melanoma tissue, 5 mm-thick representative sections were cut and placed into the array holes in an organized manner. After assembling the array, 4 μm sections were cut and mounted onto salinized glass slides. During the automated immunostaining process (Leica BOND Autostainer), sections were incubated with an anti-ACSL4 antibody (Sigma polyclonal IgG) at a 1:200 dilution for 60 min at pH 9, suitable for heat-induced epitope retrieval. Visualization employed a polymer-based Horseradish Peroxidase (HRP) system (Leica Refine HRP). To prevent the overlay of melanoma cells' internal brown pigmentation, a red colorimetric detection system was used. After drying, slides were scanned using the 3D Histech Scanner at OM 112× magnification. To quantify the intensity of the red chromogenic reaction, optical densitometry was performed in the red channel using Image-Pro Plus software. ACSL4 protein levels were measured as arbitrary optical density units in the red channel with Image-Pro Plus, following polymer-HRP detection using a red chromogen to reduce melanin interference. These values represent platform-specific densitometric units and should not be directly compared to RNA-seq abundance measurements.

## 3 Results

**Figure 1:**
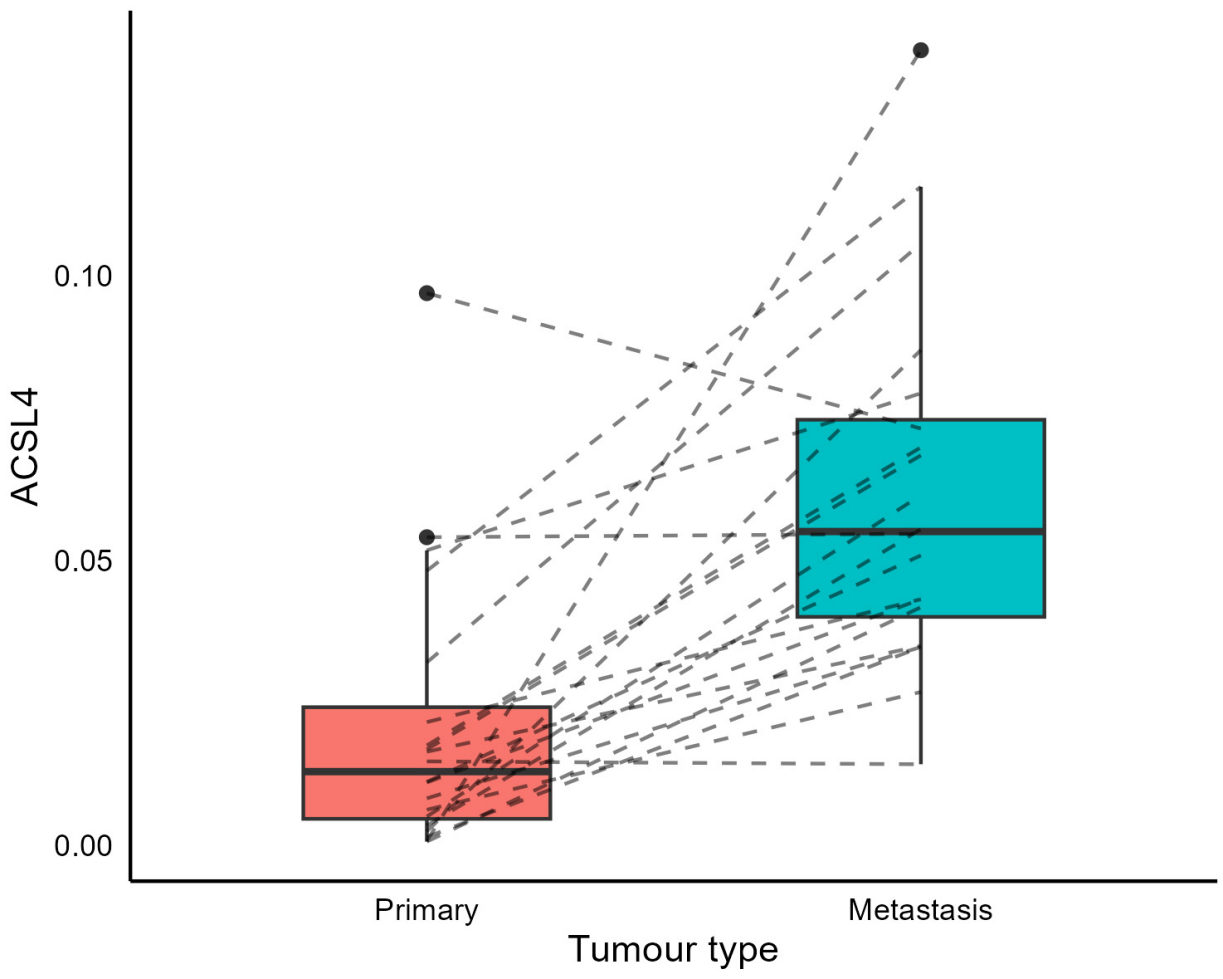
Metastatic cutaneous melanoma tumors express higher levels of ACSL4. The box-and-whisker plot compares ACSL4 protein expression levels between primary and metastatic lesions. Metastatic lesions display significantly higher ACSL4 than primary tumors in the same patients (one-sided Wilcoxon signed rank test, *p* = 1.8 × 10^−5^). Each point represents a core-level measurement; boxes indicate the interquartile range, with the median line, and whiskers span 1.5 times the interquartile range (IQR). Outliers are plotted as dots. Gray dashed lines represent the change in ACSL4 levels for an individual between primary and metastatic lesions. Scales are platform-specific (IHC optical density in this figure; TCGA normalized RNA-seq in Figure 2); absolute values are not comparable across panels.

Data from the first 20 patients included both primary and metastatic melanoma cases, along with their ACSL4 values. A significant difference in ACSL4 levels was observed (*p* = 1.8 × 10^−5^), with metastatic tumors exhibiting higher ACSL4 levels (Figure 1). This result is further confirmed in Supplementary Figure S1, which shows increased ACSL4 protein expression in metastatic melanoma compared to primary melanoma. Details on cohort selection, sampling window, and clinical descriptors are provided in Section 2.1–2.3 (Supplementary Figures S1, S2); handling of repeated IHC measures and modeling choices that account for heterogeneity are in Section 2.4, 2.6.3, 2.7, and 2.9; external validation is summarized in Section 2.8–2.11 (also shown in Supplementary Figures S3–S6).

Univariate Cox proportional hazards models were performed for DFS, MFS, and OS to examine the relationship between ACSL4 and survival in primary tumors (Supplementary Tables S3–S5). We were unable to fit models for metastatic tumors due to the limited sample size, consistent with our strategy to address cohort heterogeneity via covariate adjustment and random-effects survival rather than underpowered subgrouping (Section 2.2–2.4, 2.6.3, 2.7, 2.9), with external replication in TCGA/TIDE/TIMER (Sections 2.8–2.11; Supplementary Figures S1–S3, S6). Supplementary Table S3 displays the univariate results specifically for DFS. We present the univariate analysis for DFS, the survival variable with the most significant number of practical observations, that is, the least number of censorings first, and we follow the same order for the rest of the results. For DFS, ACSL4 emerges as a significant predictor. Several other covariates, such as age, appear to be nominally significant. Patients with higher ACSL4 values had lower hazard rates and higher survival rates. We also created Kaplan–Meier curves to illustrate the relationship between ACSL4 and DFS. As ACSL4 is a continuous variable, we divided our data into two groups based on the median ACSL4 values. Supplementary Figure S2 shows the Kaplan–Meier curves for DFS, indicating the association of higher ACSL4 levels with longer survival durations.

Supplementary Table S4 shows the univariate results for MFS. ACSL4 was not a statistically significant predictor, but the number of metastatic localizations was. Supplementary Table S5 presents the univariate results for OS. The number of metastatic localizations again appears as a significant predictor, Breslow thickness is nominally significant, and ACSL4 remains not significantly associated.

Univariate analysis may not capture the full picture due to relationships or confounding factors among variables. Therefore, we examined the impact of ACSL4 through a multivariate analysis across all three survival outcomes. A model selection process was performed using the Akaike Information Criterion (AIC) to assess a set of candidate models. This multivariable framework, combined with random-effects survival analysis for repeated measurements and study-wise BKY FDR control (Sections 2.7 and 2.9), addresses cohort heterogeneity without fragmenting the modest sample into underpowered subgroups; external replication is provided in Sections 2.8–2.11. Tables 1–3 present the final multivariate model for DFS, MFS, and OS. In the multivariable OS model derived from the Szeged primary melanoma cohort (Table 3), higher ACSL4 expression remained independently associated with a lower hazard of death after adjustment for the number of metastatic localizations, age at diagnosis, and sex. Thus, in this single-center IHC dataset, ACSL4 is a significant predictor across all three survival endpoints (DFS, MFS, and OS). The β-coefficients for ACSL4 in these models were negative, indicating that higher levels of ACSL4 were associated with more prolonged survival.

**Table 1:** Multivariate model for disease-free survival (DFS).

**Variable**	**β**	**HR [95% CI for HR]**	***p*-value**
ACSL4	−19.1000	5.07e-09 [1.71e-14–1.51e-03]	**0.003**
Loc_m_cnt	0.3107	1.364 [1.10–1.70]	**0.006**
Age	0.0303	1.031 [0.99–1.07]	0.116
Sex	0.5648	1.759 [0.72–4.29]	0.215

Cox proportional hazards models including ACSL4 expression, number of metastatic localizations (Loc_m_cnt), age at diagnosis, and sex. Reported for the table are β (log-hazard) coefficients, hazard ratios (HR) with 95% confidence intervals, and Wald p-values. Models were selected based on the Akaike Information Criterion (AIC) and account for repeated ACSL4 measurements per patient through a patient-level random-effects (frailty) term. Across all three outcomes, ACSL4 shows a negative β (HR < 1), indicating an association with longer survival, while Loc_m_cnt, defined as the count of distinct metastatic sites, has a positive β, indicating a higher hazard. The p-values are two-sided; where applicable, study-wise false discovery rate was controlled using the Benjamini–Krieger–Yekutieli (BKY) procedure.

HR, hazard ratio; CI, confidence interval. Values shown in bold indicate *p*-values that were regarded as statistically significant.

**Table 2:** Multivariate model for metastasis-free survival (MFS).

**Variable**	**β**	**HR [95% CI for HR]**	***p*-value**
ACSL4	−16.0500	1.07e-07 [2.67e-12–4.25e-03]	**0.003**
Loc_m_cnt	0.4789	1.614 [1.29–2.03]	**3.8e-5**
Age	0.0198	1.020 [0.98–1.06]	0.287
Sex	0.7027	2.019 [0.86–4.74]	0.106

Cox proportional hazards models including ACSL4 expression, number of metastatic localizations (Loc_m_cnt), age at diagnosis, and sex. Reported for the table are β (log-hazard) coefficients, hazard ratios (HR) with 95% confidence intervals, and Wald p-values. Models were selected based on the Akaike Information Criterion (AIC) and account for repeated ACSL4 measurements per patient through a patient-level random-effects (frailty) term. Across all three outcomes, ACSL4 shows a negative β (HR < 1), indicating an association with longer survival, while Loc_m_cnt, defined as the count of distinct metastatic sites, has a positive β, indicating a higher hazard. The p-values are two-sided; where applicable, study-wise false discovery rate was controlled using the Benjamini–Krieger–Yekutieli (BKY) procedure.

HR, hazard ratio; CI, confidence interval. Values shown in bold indicate *p*-values that were regarded as statistically significant.

**Table 3:** Multivariate model for overall survival (OS).

**Variable**	**β**	**HR [95% CI for HR]**	***p*-value**
ACSL4	−16.6300	5.99e-08 [1.38e-12–0.003]	**0.002**
Loc_m_cnt	0.4970	1.644 [1.30–2.07]	**2.6e-5**
Age	0.0210	1.021 [0.98–1.06]	0.280
Sex	0.4485	1.566 [0.69–3.53]	0.280

Cox proportional hazards models including ACSL4 expression, number of metastatic localizations (Loc_m_cnt), age at diagnosis, and sex. Reported for the table are β (log-hazard) coefficients, hazard ratios (HR) with 95% confidence intervals, and Wald p-values. Models were selected based on the Akaike Information Criterion (AIC) and account for repeated ACSL4 measurements per patient through a patient-level random-effects (frailty) term. Across all three outcomes, ACSL4 shows a negative β (HR < 1), indicating an association with longer survival, while Loc_m_cnt, defined as the count of distinct metastatic sites, has a positive β, indicating a higher hazard. The p-values are two-sided; where applicable, study-wise false discovery rate was controlled using the Benjamini–Krieger–Yekutieli (BKY) procedure.

HR, hazard ratio; CI, confidence interval. Values shown in bold indicate *p*-values that were regarded as statistically significant.

**Figure 2:**
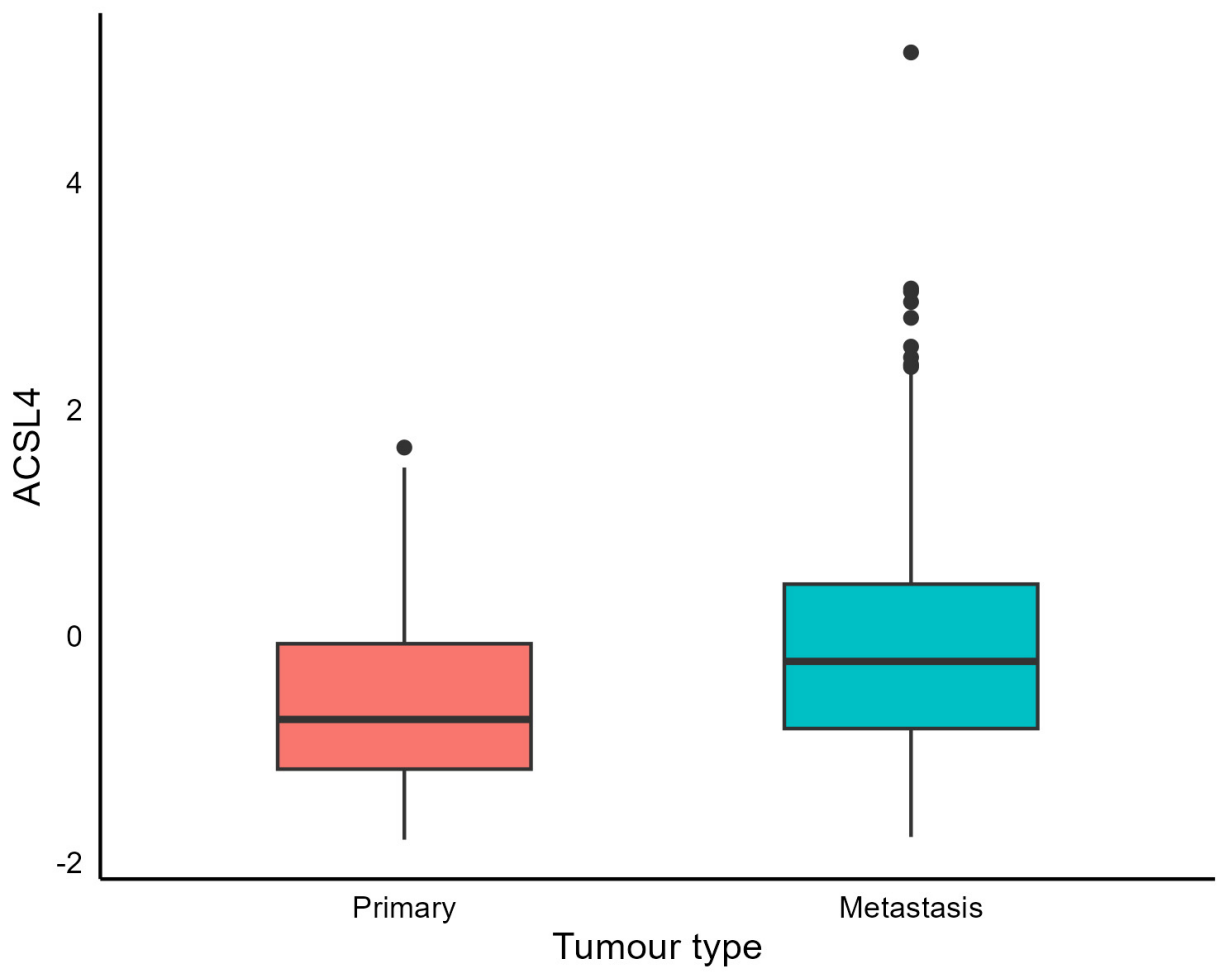
Metastatic cutaneous melanoma tumors express high ACSL4 levels. Boxplots compare ACSL4 expression in TCGA–Skin Cutaneous Melanoma (SKCM) primary (*n* = 109) and metastatic (*n* = 371) tumors. Center line shows the median; box, interquartile range (IQR); whiskers, 1.5 × IQR; points, outliers. ACSL4 expression is significantly higher in metastases (linear regression adjusted for age and sex, *p* = 1.3 × 10^−5^). Scales differ by platform (IHC optical density in Figure 1; TCGA normalized RNA-seq in this figure); absolute values cannot be directly compared across panels.

We conducted a similar analysis on the considerably larger dataset from The Cancer Genome Atlas (TCGA), comprising 480 records. Here, we also demonstrated that ACSL4 levels in metastatic tumors were significantly higher than those in primary tumors (*p* = 1.3 × 10^−5^). The accompanying boxplot is provided in Figure 2.

As discussed previously, we fitted multivariate CoxPH models for each of the two groups (primary and metastatic tumors). It is important to note that the TCGA dataset did not include information on MFS; only OS and DFS were included. In primary tumors, ACSL4 emerged as a significant predictor of DFS but not of OS. However, for metastatic tumors, ACSL4 was observed to be an important predictor for both survival outcomes (DFS and OS). Table 4 summarizes the analysis results from the TCGA dataset. We observe that the TCGA results differ from those of the Szeged cohort regarding overall survival (OS) in primary tumors. Although ACSL4 shows a significant association with OS in the Szeged multivariable model (Table 3), it does not reach significance in the TCGA primary-tumor subset (Table 4). This discrepancy likely stems from differences in cohort composition and available information: the TCGA primary group (*n* = 109) is enriched for earlier-stage lesions, has relatively few deaths, and exhibits substantial right censoring. Additionally, TCGA models include only ACSL4, age, and sex, while Szeged models also account for metastatic burden (Loc_m_cnt) and include a patient-level random-effects term to leverage multiple ACSL4 measurements per tumor.

**Table 4:** Survival Analysis for overall survival (OS) and metastasis-free survival (MFS) in the TCGA dataset.

**Tumor type**	**Survival outcome**	***p*-Value for ACSL4**
Primary	MFS	**0.001**
	OS	0.738
Metastasis	MFS	**0.005**
	OS	**2E-5**

Summary of ACSL4 correlations with survival in the TCGA SKCM cohort (*n* = 480), divided by tumor type (primary vs. metastatic). The p-values for the ACSL4 variable from multivariate Cox models, adjusted for age and sex, were calculated separately for primary (*n* = 109) and metastatic tumors (*n* = 371). Significance was determined using study-wide FDR control (Benjamini-Krieger-Yekutieli). In metastatic tumors, ACSL4 is significant for both overall survival (OS) and metastasis-free survival (MFS); in primary tumors, it is significant only for MFS, not OS.

OS, overall survival; MFS, metastasis-free survival. Values shown in bold indicate *p*-values that were regarded as statistically significant.

**Figure 3:**
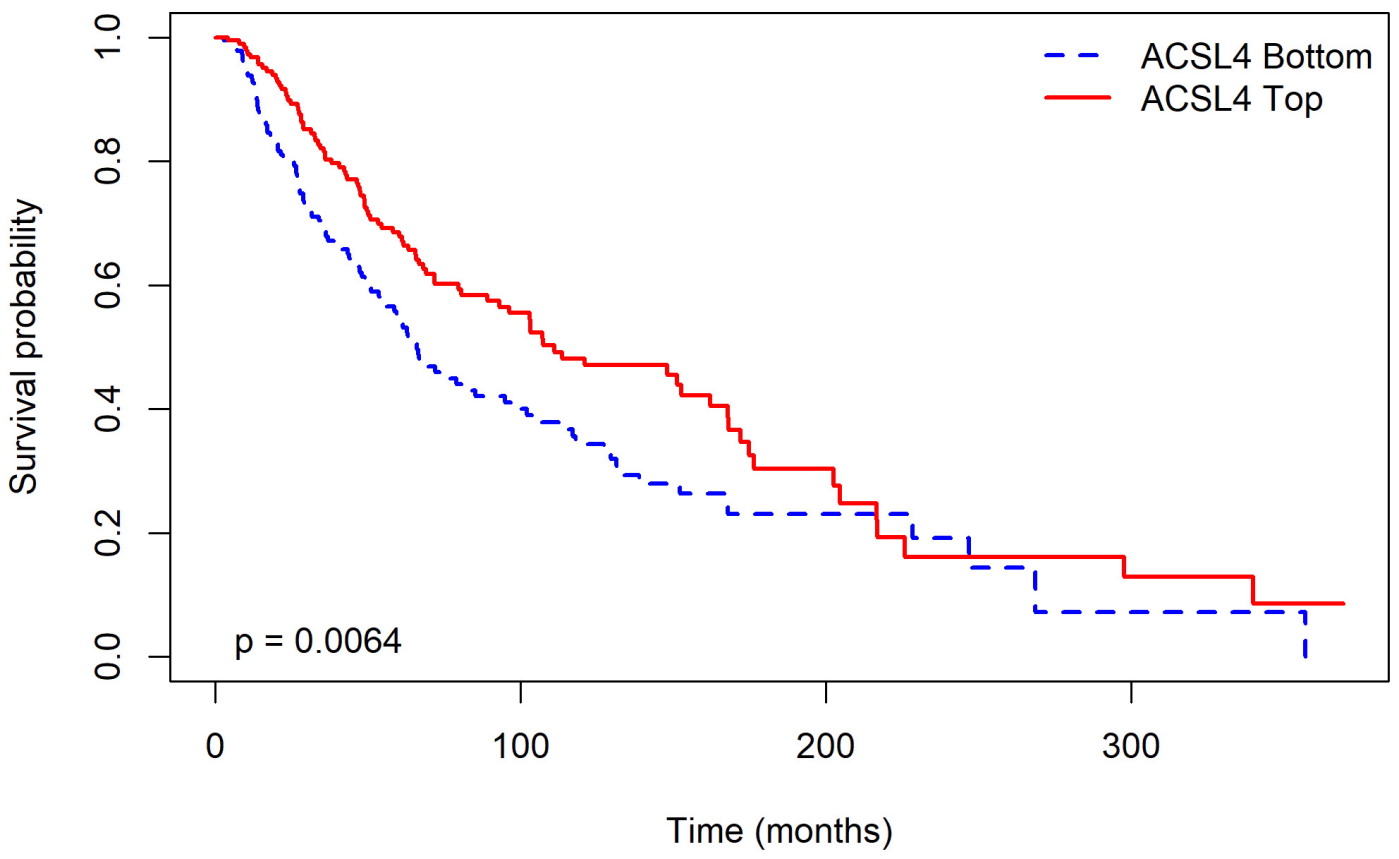
ACSL4 emerges as a significant predictor of survival probability in cutaneous melanoma tumors. Kaplan–Meier curves display overall survival (OS) in TCGA metastatic SKCM cases, divided by median ACSL4 expression levels. Patients with elevated ACSL4 levels tend to have longer OS, with a log-rank *p*-value of 0.0064. Tick marks indicate censored data points; time is measured in months. The grouping and statistical analysis follow the methods described, using the Kaplan–Meier estimator and log-rank test.

Figure 3 shows the Kaplan–Meier curve for OS in metastatic tumors, demonstrating a positive association with more prolonged survival, consistent with the observations in our initial dataset. As ACSL4 is a continuous variable, we divided our data into two groups based on the median ACSL4 values.

In conclusion, our analysis of the TCGA dataset, which included a much larger sample size of 480 records, produced results consistent with our earlier findings from the smaller dataset. These consistent TCGA results directly address the issue of generalizability beyond the initial discovery setting. Therefore, they offer further validation and strengthen our observations. Specifically, we found that ACSL4 consistently emerged as a significant predictor for OS and DFS. Notably, higher levels of ACSL4 were linked to longer survival times.

To confirm our results, we also examined Kaplan–Meier curves from the Tumor Immune Dysfunction and Exclusion (TIDE) website, utilizing datasets from multiple prior studies. Supplementary Figure S3 shows the relationship between ACSL4 and MFS according to the Gide et al. _PD1+CTLA4 study (21). Interestingly, patients in the “ACSL4 Top” group seem to have a longer MFS in this analysis.

We further reviewed the Lauss et al. ACT study (22), finding similar patterns in how ACSL4 relates to survival outcomes. Patients classified as “ACSL4 Top” showed increased overall survival (OS) and metastasis-free survival (MFS), as detailed in Supplementary Figure S4. We also analyzed the GSE8401 study (23), where we observed consistent results linking ACSL4 expression to OS. Similarly, in the GSE54467 study, patients in the “ACSL4 Top” group again demonstrated longer OS, as illustrated in Supplementary Figure S5.

Finally, the relationship between ACSL4 expression and the invasion of tumor immune-infiltrating cells (TIICs) was examined. Notably, the positive associations between ACSL4 expression and TIIC estimates (B cells, CD8^4^ and CD4^4^ T cells, macrophages, neutrophils, dendritic cells) persisted after tumor-purity adjustment within TIMER and remained consistent under study-wide BKY FDR control (Supplementary Figure S6). Thus, these results collectively indicate that ACSL4 expression is positively linked to TIICs, which may influence how patients with these tumors respond to immune checkpoint inhibitors (ICIs).

## 4 Discussion

This study focused on exploring the role of ACSL4 expression in cutaneous melanoma cells by analyzing two datasets and comparing their results. To address generalizability, we replicated the principal associations in TCGA (*n* = 480) and observed consistent direction and significance. We also corroborated survival patterns via TIDE and immune-infiltration correlations using TIMER. Together with AIC-guided multivariable modeling and FDR control in the discovery cohort, these analyses mitigate concerns related to the single-center sample (Sections 2.8–2.11 and 3, Figures 2, 3; Table 4; Supplementary Figures S3–S6). Overall, the findings suggest that ACSL4 expression in cutaneous melanoma is associated with improved survival rates. Elevated ACSL4 levels were observed in metastatic melanoma cells compared with non-metastatic cells. Cells with higher ACSL4 expression showed increased immune cell infiltration compared to cells with lower levels. Together, these results suggest that higher ACSL4 expression may improve cutaneous melanoma outcomes. Therefore, examining ACSL4 expression could provide valuable insights into melanoma cell behavior and inform potential therapeutic decisions.

The different *y*-axis ranges in Figures 1, 2 reflect differences across platforms and cohorts: IHC densitometry measures protein levels in specific tumor regions, while TCGA bulk RNA-seq assesses mRNA levels in samples with varying tumor purity and microenvironmental factors. Additional variability arises from differences in sample collection and processing across cohorts, as well as repeated core-level measures in the discovery TMA vs. single bulk profiles in TCGA. These factors are expected to impact absolute distributions but do not compromise the within-dataset comparisons. Notably, both platforms show higher ACSL4 levels in metastases (Wilcoxon *p* < 0.0001 for each), and ACSL4 remains independently associated with better survival in multivariable analyses and in external validation, underscoring the robustness of the biological signal despite platform differences.

Previous studies have investigated how specific tumor features, in combination with ACSL4 expression, affect melanoma cells' responses to immune-targeting therapies. Liu et al. analyzed a cohort of 144 melanoma patients treated with anti-PD1 immune checkpoint blockade (ICB) by performing whole-exome and whole-transcriptome sequencing on pre-treatment tumors. Their findings indicated that the effectiveness of anti-PD1 ICB depended on the melanoma subtype, with responses varying according to genomic and transcriptomic characteristics (24). Additionally, Liu et al. demonstrated that melanoma cells initially responding well to ICB can develop various resistance mechanisms over time, ultimately leading to late recurrence (25).

The response of melanoma cells to monoclonal antibodies directed against cytotoxic T-lymphocyte-associated protein 4 [CLTA-4] was also investigated. Van Allen et al. analyzed whole exomes from pre-treated melanoma biopsies in 110 patients. They were unable to identify any specific peptide sequence that predicted response to this immune checkpoint inhibitor, emphasizing the need for further studies, possibly with larger sample sizes (26). Riaz et al. (27) also studied genomic changes in 68 patients with advanced melanoma that either progressed despite ipilimumab (a monoclonal antibody against CTLA-4) treatment or never received ipilimumab. These patients were then treated with nivolumab (a programmed death-1 blocking antibody). In patients who responded to nivolumab, tumor cells showed activation of specific transcriptional networks and increased expression of immune checkpoint genes (27). This indicates that tumor cells undergo immunoediting through a therapy-dependent mechanism that shapes their response to treatment.

ACSL4 expression is yet another tumor feature that can be better understood to determine its effect on tumor growth and response to therapy. This is especially important given that prior studies have yielded conflicting data on the effect of ACSL4 expression, with results varying by cancer type (6). Thus, ACSL4 expression may be beneficial or harmful depending on the type of cancer, which necessitates further studies to elucidate its role in individual cancer subtypes.

In the present study, the results suggest that ACSL4 expression may play an essential role in tumor cells' response to immune checkpoint inhibitors (ICIs). ICIs promote T-cell-mediated ferroptosis (28). As discussed above, ACSL4 enhances sensitivity to ferroptosis, thereby increasing the likelihood of ferroptosis. Therefore, it is plausible that higher ACSL4 expression enhances responsiveness to immune checkpoint inhibitors (ICIs). However, this remains a hypothesis: our study did not evaluate ICI response, and the observed associations, higher ACSL4 with more prolonged survival and greater immune infiltration, are correlative. Thus, any claim that ACSL4-driven ferroptosis enhances ICI efficacy should be viewed as a testable hypothesis rather than a conclusion, as this study did not experimentally assess treatment response. Prospective, mechanistic studies are needed to establish causality.

The data also show that patients with increased ACSL4 expression exhibited significantly improved OS, despite higher ACSL4 levels in metastatic melanoma cells. Melanoma can metastasize through blood vessels, lymphatic vessels, or both. Certain features of melanoma can predict whether it spreads via the blood or the lymphatic system. In a study of 1,177 patients (29), older age (>55 years), primary tumor location in the head or neck, increased Breslow thickness (>4.00 mm), and vascular invasion were linked to lymphatic spread, while increased Breslow thickness (>4.00 mm), lack of regression, TERT promoter mutations, and BRAF mutations related to blood-borne spread. In that study, an equal percentage of patients showed either lymphatic or blood spread in cases of metastasis, indicating that one route is not necessarily more common than the other. Both blood and lymphatic spread were observed simultaneously, suggesting that tumor cells may possess traits that increase the likelihood of both, leading to the development of simultaneous metastases (29). Mechanistically, ACSL4 enhances sensitivity to ferroptosis, and prior work suggests that circulating melanoma cells in the blood experience greater oxidative and ferroptosis stress than those transiting through lymphatic vessels (29). Accordingly, high ACSL4 expression, particularly in the context of hematogenous spread, could enhance ferroptosis-mediated elimination of tumor cells and improve survival. However, our dataset did not classify metastases by route; future studies stratified by dissemination pathway are warranted to test this hypothesis.

Several limitations of this study should be noted. First, differences such as cross-platform and cohort heterogeneity, the use of protein IHC on TMAs vs. bulk RNA-seq in TCGA, and variable tumor purity and sample processing likely account for the variations in ACSL4 distributions. These factors prevent a direct comparison of absolute values across figures, even though the within-platform contrasts are consistent. Next, there is always the possibility that other confounding variables may influence the results that were not accounted for in the assessment methods. Also, the initial dataset had a relatively small sample size, especially considering that survival data, by nature, have even fewer effective observations due to censoring. This could be the reason why DFS, the variable with the most significant number of effective observations, was the only variable that showed a significant association with ACSL4 in the univariate analysis. This could also explain why OS, the variable with the fewest effective observations, did not show a significant link with primary tumors in the TCGA data. Although the results from the initial dataset were validated by those from larger datasets, it would be ideal to further validate the findings by repeating the analyses using even larger datasets with detailed covariates to account for potential heterogeneity, thereby increasing confidence in the reliability and validity of the results. Nevertheless, the uniform single-center workflow and concordant validations in TCGA, TIDE, and TIMER support the robustness and generalizability of the main findings.

An important nuance or limitation is the apparent discrepancy between the Szeged IHC cohort and the TCGA primary-tumor subset regarding OS. In our single-center discovery cohort, ACSL4 remained an independent predictor of improved OS in multivariable modeling (Table 3). In contrast, in TCGA primary tumors, ACSL4 was significantly associated with MFS but not with OS, whereas it retained significance for both endpoints in metastatic tumors (Table 4). We interpret this difference as a result of cohort design and statistical power rather than a true biological inconsistency. The TCGA primary subset is relatively small (*n* = 109) and enriched for earlier-stage lesions with limited death events and extensive right censoring, which reduces power to detect OS effects compared with DFS. Additionally, the Szeged models incorporate metastatic burden (Loc_m_cnt) and a frailty term to account for repeated IHC measurements, whereas the TCGA models are necessarily limited to ACSL4, age, and sex. Taken together, the consistent direction of association across endpoints and the strong significance of ACSL4 in our discovery cohort and in metastatic TCGA cases support the conclusion that higher ACSL4 expression is linked to better outcomes.

While TIMER provides robust, cohort-scale TIIC estimates from bulk expression, deconvolution has inherent constraints. First, lineage granularity is limited to major immune classes, which may not resolve functional states or spatial context. Second, tumor purity estimation and cross-sample heterogeneity can introduce residual bias even after partial correlation adjustment. Third, inferred abundances depend on reference marker sets and bulk RNA-seq preprocessing, which can vary across datasets. We therefore interpret ACSL4-TIIC associations as correlative; they support biological plausibility alongside our survival findings but do not establish causality. Future single-cell or spatial profiling studies will be needed to validate cell-state-specific infiltration patterns. Finally, this study uses retrospective datasets, which may not be representative of the entire population, potentially introducing selection bias. It is also possible that retrospective data has limitations in data quality, such as variable accuracy in the recorded information.

In conclusion, ACSL4 is a well-established driver of ferroptosis. In melanoma, our data suggest that variation in ACSL4 expression is associated with features relevant to prognosis; however, these observations are correlative and, by themselves, do not establish causality. To move from association to mechanism, we propose: (1) genetic gain- and loss-of-function of ACSL4 in melanoma models with readouts of lipid peroxidation and ferroptosis-mediated cell death (e.g., C11-BODIPY), including rescue by ferroptosis inhibitors; (2) co-culture assays with CD8^4^ T cells and/or exposure to immune checkpoint inhibitors (ICIs) to test whether ACSL4 modulation alters T-cell-mediated ferroptosis; and (3) *in vivo* studies in syngeneic melanoma to assess whether ACSL4 manipulation affects tumor growth, ferroptosis markers, and the composition and spatial organization of tumor-infiltrating immune cells, complemented by single-cell or spatial transcriptomics.

The proposed studies, while beyond the scope of this work, would thoroughly determine whether ACSL4 causally links ferroptosis to antitumor immunity in melanoma. Therefore, mechanistic experiments to establish the ACSL4-ferroptosis axis and treatment-response evaluations were intentionally excluded from this retrospective, association-focused manuscript and are warranted in future dedicated functional and *in vivo* studies.

## 5 Conclusion

In summary, the findings suggest that ACSL4 expression in melanoma cells promotes survival, likely by inducing ferroptosis-mediated cell death, and is also elevated in metastatic cells. Future studies should focus on understanding how ACSL4 influences survival rates among melanoma patients with metastases, considering the different types of metastases. Moreover, additional studies could explore how ACSL4 influences immune cell infiltration into tumors and the mechanisms behind this process's role in promoting tumor cell death. Once its function in melanoma is fully clarified, ACSL4 expression might serve as a prognostic marker to assist patient counseling and could be targeted in therapies designed to modify its levels.

## Data Availability

The raw data supporting the conclusions of this article will be made available by the authors, without undue reservation.
