# Supplementary material for: Acyl-CoA synthetase long-chain family member 4—A novel prognostic marker in cutaneous melanoma
Source: Front Med (Lausanne). 2026 Mar 16;12:1553961. doi: 10.3389/fmed.2025.1553961 (PMC13035052; doi:10.3389/fmed.2025.1553961)
Supplement: Supplementary file 1 [file Data_Sheet_1.docx]

Supplementary Information

Acyl-CoA Synthetase Long Chain Family Member 4 (ACSL4):
A Novel Prognostic Marker in Cutaneous Melanoma

**Soumya Paria^1¶^, Rebecca Lapides^2,3¶^, Babak Saravi^4^, Anjali Rajagopal^5^, Alina M. Mueller^6^,
Peter Kölblinger^7^, Michael Wang-Evers^3^, Dieter Manstein^3^, Alexander A. Navarini^6^, Peter Lazar^8^, Lajos Kemény^9,10,11^, Grzegorz Sarek^6^, Kaustubh Adhikari^1,12*^, István B. Németh^13*^, Elisabeth Roider^3,6*^**

^1^ School of Mathematics and Statistics, Faculty of Science, Technology, Engineering and Mathematics, The Open University, Milton Keynes, MK7 6AA, United Kingdom

^2^ The Robert Larner, M.D. College of Medicine at the University of Vermont, Burlington, Vermont, United States of America

^3^ Cutaneous Biology Research Center, Department of Dermatology, Massachusetts General Hospital, Harvard Medical School, Charlestown, Massachusetts, United States of America

^4^ Department of Orthopedics and Trauma Surgery, Medical Center, University of Freiburg, Germany

^5^ Department of Internal Medicine, University of Connecticut, Farmington, Connecticut, United States of America

^6^ Department of Dermatology, University Hospital of Basel, Basel, Switzerland

^7^ Department of Dermatology and Allergology, Paracelsus Medica University, Salzburg, Austria

^8^ Department of Oral and Maxillofacial Surgery, Medical Center, University of Szeged, Hungary

^9^ Department of Dermatology, Venereology and Dermatooncology, Faculty of Medicine, Semmelweis University, Budapest 1085, Hungary

^10^ HCEMM-SU Translational Dermatology Research Group, Semmelweis University, Budapest 1094, Hungary

^11^ Department of Physiology, Semmelweis University, Budapest 1094, Hungary

^12^ Department of Genetics, Evolution and Environment, and UCL Genetics Institute, University College London, London WC1E 6BT, United Kingdom

^13^ Department of Dermatology and Allergology, Szent-Györgyi Albert Medical School,
University of Szeged, 6720 Szeged, Hungary

^¶^These authors contributed equally to this work.

*** Correspondence:**Kaustubh Adhikari; E-mail: [kaustubh.adhikari@open.ac.uk](mailto:kaustubh.adhikari@open.ac.uk)

István Németh; E-mail: [nemethistvanbalazs@gmail.com](mailto:nemethistvanbalazs@gmail.com)

Elisabeth Roider; E-mail: [elisabeth.roider@usb.ch](mailto:elisabeth.roider@usb.ch)

*
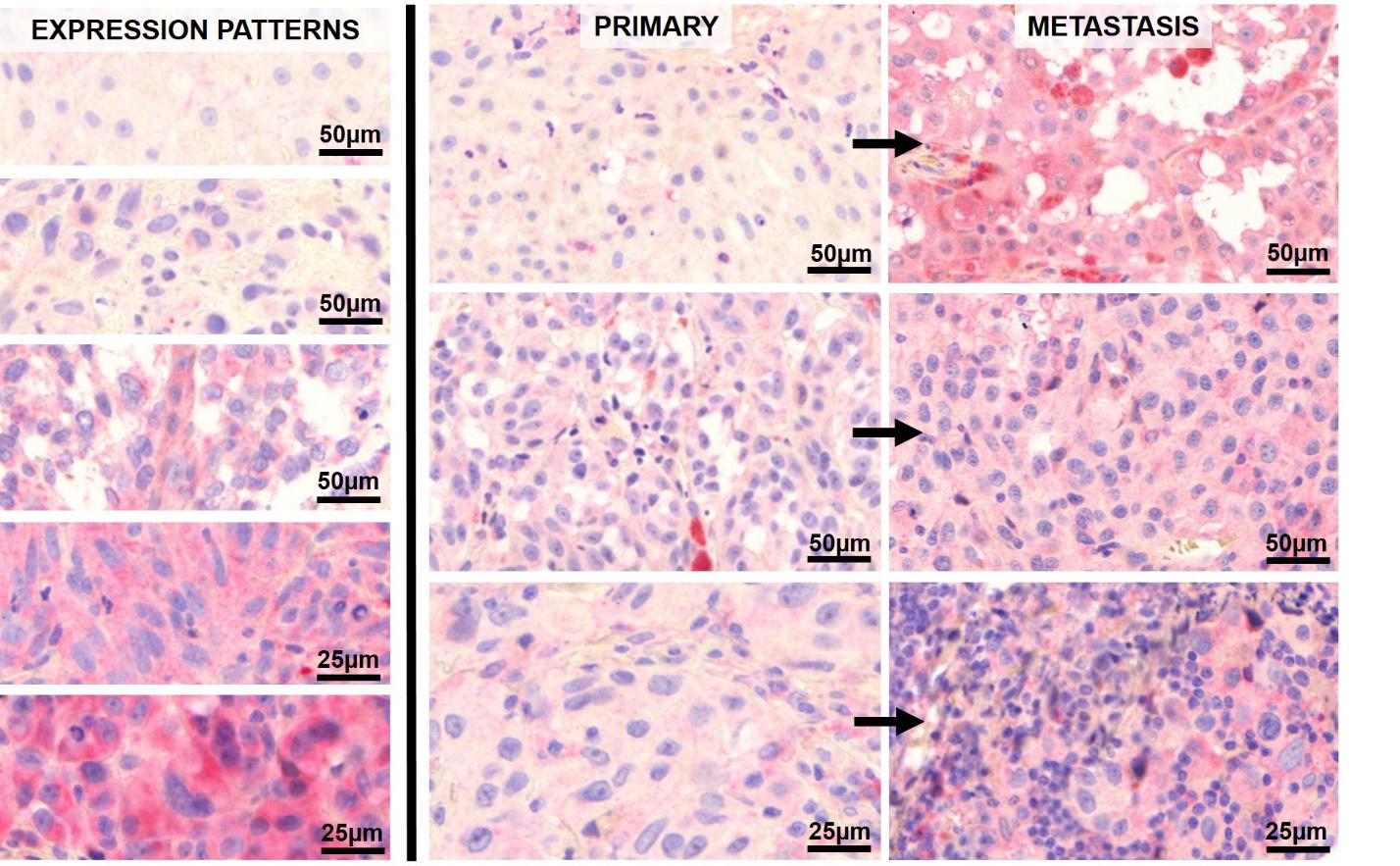
*

**Supplementary Figure S1**: **ACSL4 protein expression in melanoma.** Left insets: representative immunohistochemistry (IHC) illustrating a staining spectrum from negative (top) through focal to diffuse with increasing intensity (bottom). Right panels: paired comparison of ACSL4 expression between primary melanomas and their matched metastases (three patient pairs).


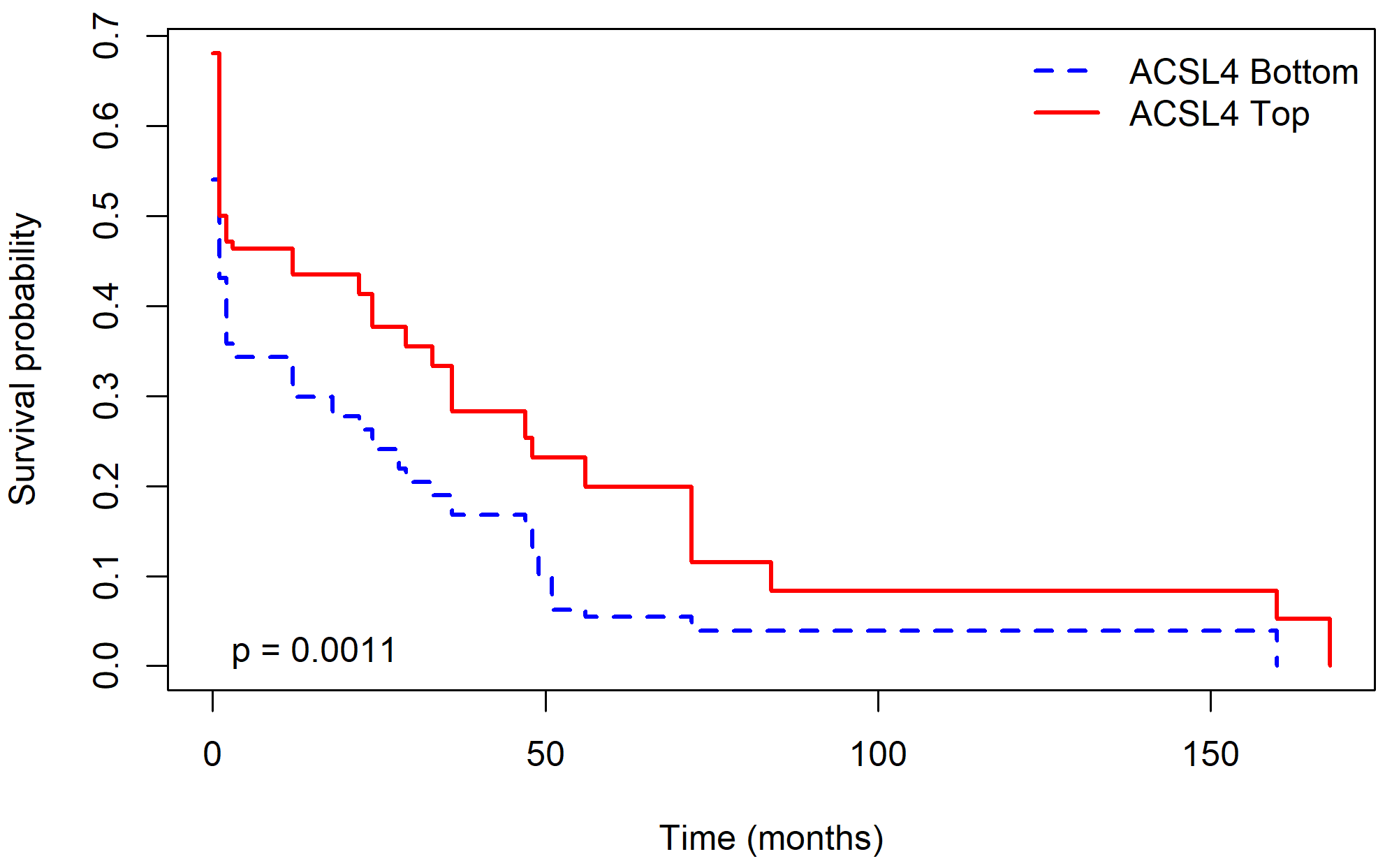


**Supplementary Figure S2. ACSL4 expression associates with prolonged disease-free survival in cutaneous melanoma.** Kaplan-Meier curves for DFS in the patient cohort, stratified into ACSL4-High and ACSL4-Low groups by a median split of tumor ACSL4 expression. Time zero is the date of primary melanoma diagnosis; DFS is defined as the interval from diagnosis to the first subsequent disease event. Step functions display Kaplan-Meier estimates with 95% confidence intervals. Tick marks denote censored data, and the numbers at risk are displayed beneath the x-axis (where shown). Group differences were assessed with a two-sided log-rank test. Higher ACSL4 expression tracks with longer DFS, consistent with the multivariable Cox models reported in the main text.


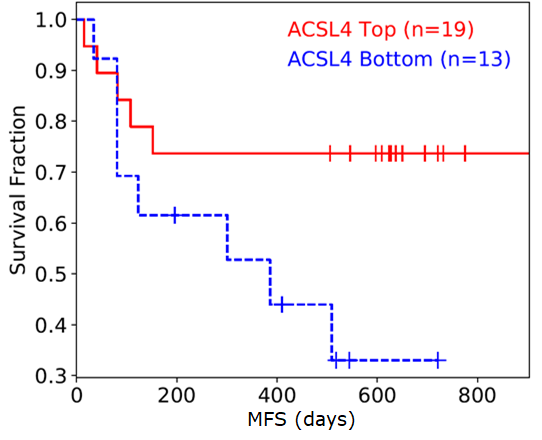


**Supplementary Figure S3. Kaplan-Meier curves for the Gide et al., PD1+CTLA4 study [21].** Kaplan-Meier analysis of **metastasis-free survival (MFS)**in the Gide et al. cohort treated with anti-PD-1 monotherapy or combined anti-PD-1/anti-CTLA-4 (PD1+CTLA4), stratified by tumor **ACSL4 e**xpression. Tick marks indicate censored data. Groups were compared by a two-sided log-rank test, with hazard ratios estimated using a univariate Cox model. In this cohort, higher ACSL4 expression (Top) is associated with longer MFS.


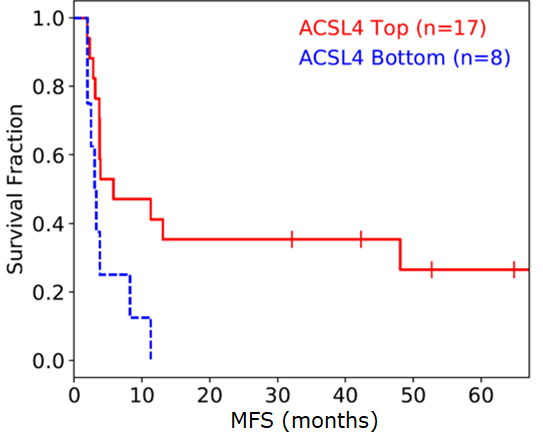


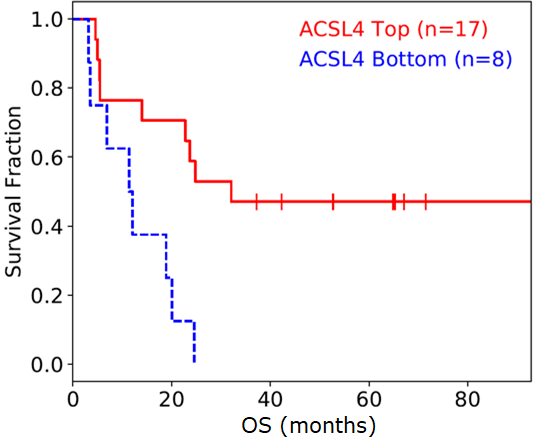


**Supplementary Figure S4. Kaplan–Meier curves for the Lauss et al., study** **[22].** Overall survival (OS) and metastasis-free survival (MFS) in the Lauss et al., [22] adoptive cell therapy cohort, stratified by ACSL4 expression (ACSL4 Top vs ACSL4 Bottom). Patients were divided into groups based on the median ACSL4 level, as implemented on the TIDE resource.
Curves depict the survival probability over time (in months), with tick marks indicating censored data. Log-rank p-values are shown in each panel. Consistent with our external validation, the “ACSL4 Top” group shows longer OS and MFS than the “Bottom” group.


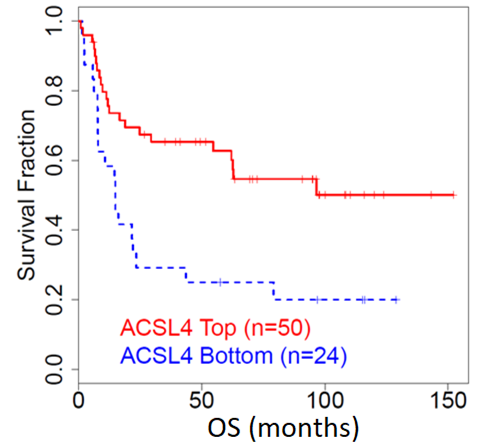


**Supplementary Figure S5. Kaplan-Meier curves for the GSE54467 study [22].** Overall survival (OS) in the GSE54467 melanoma cohort [22], stratified by ACSL4 expression (ACSL4 Top vs ACSL4 Bottom) using the TIDE resource. Patients were divided into two groups based on the median ACSL4 level; curves display survival probability over time, with log-rank p-values reported by TIDE. Consistent with our external validation, the “ACSL4 Top” group exhibits longer OS.


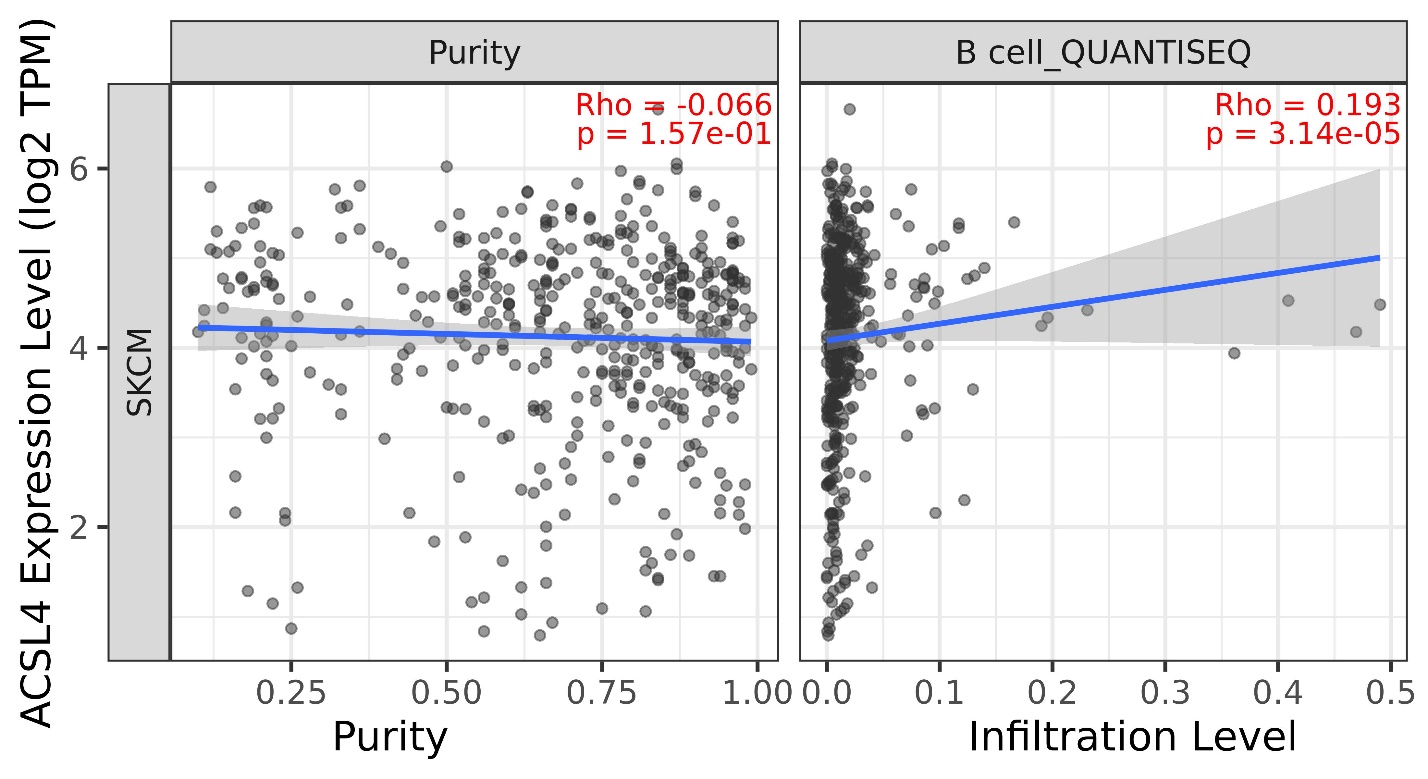


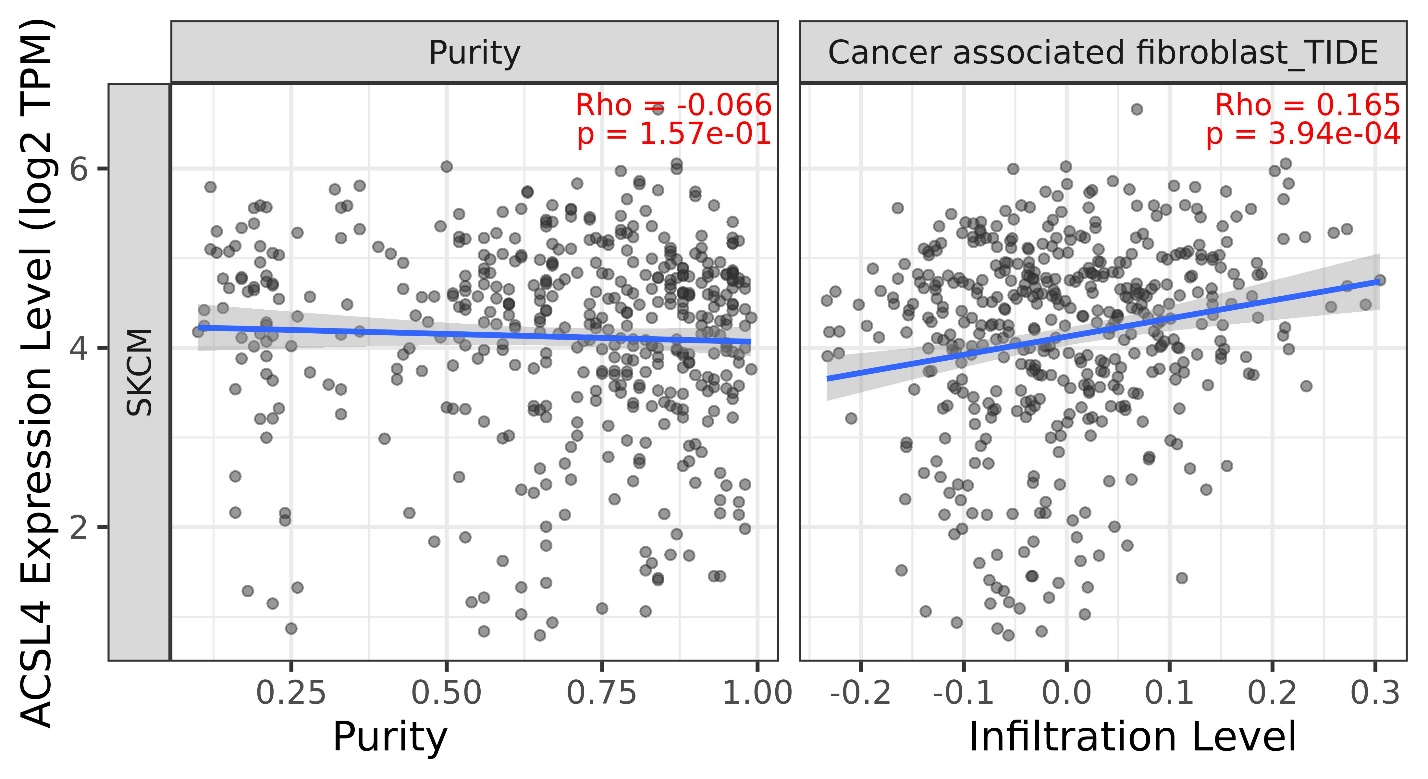


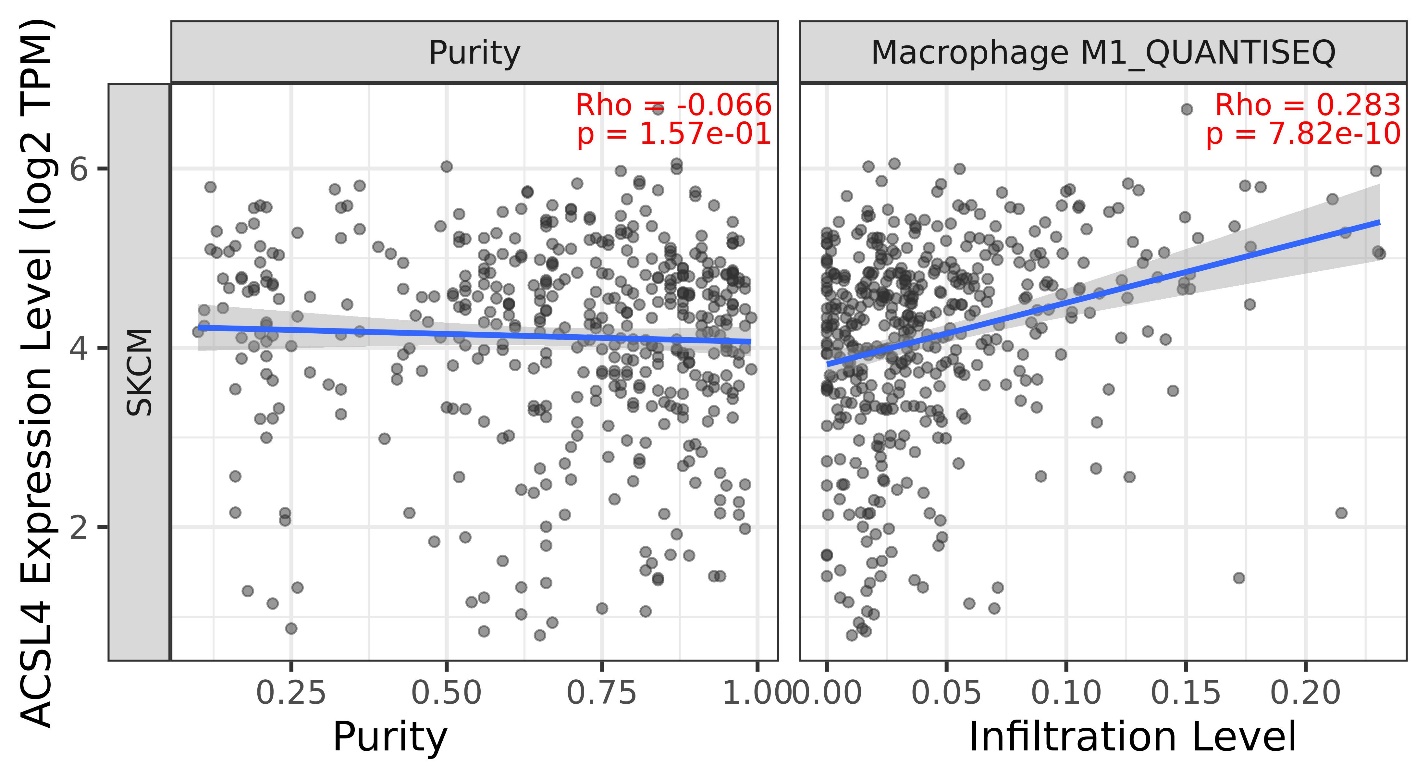


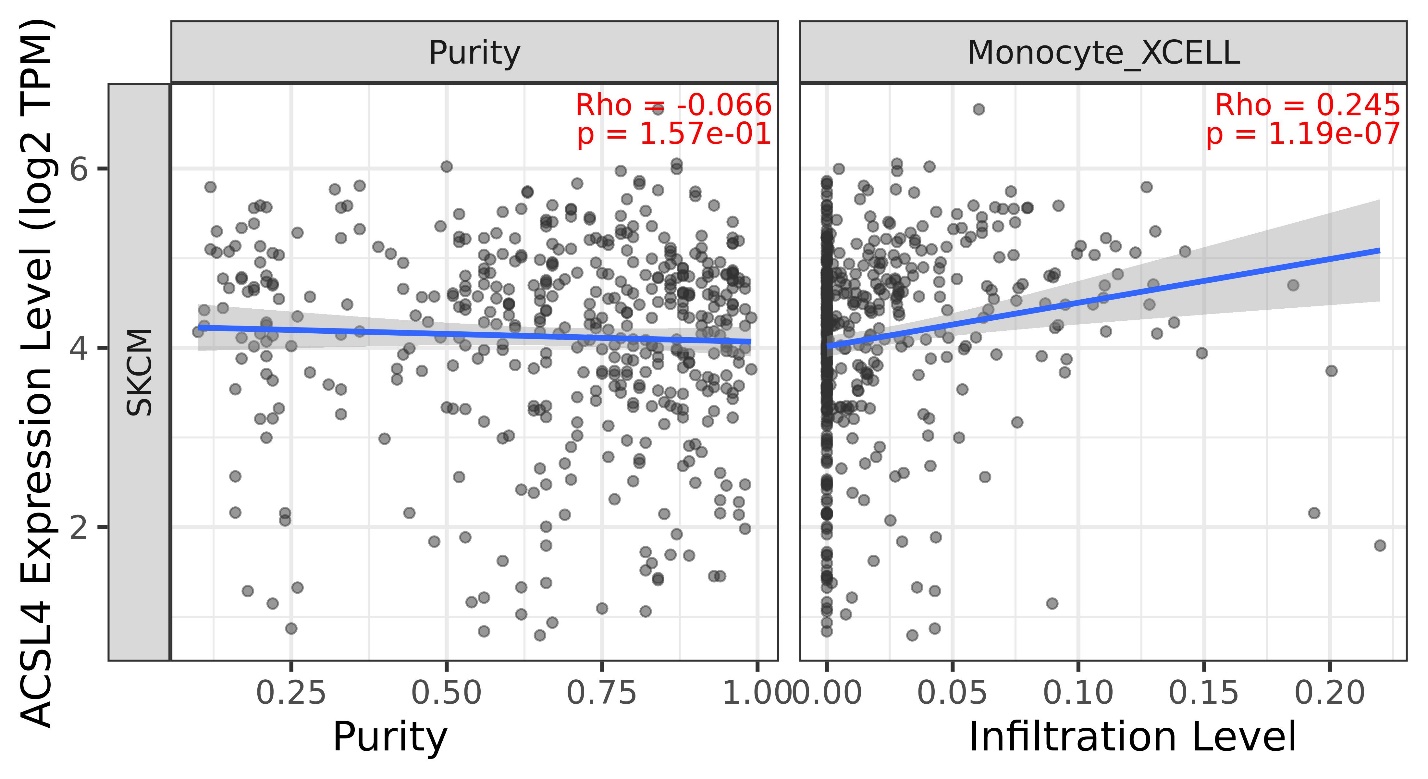


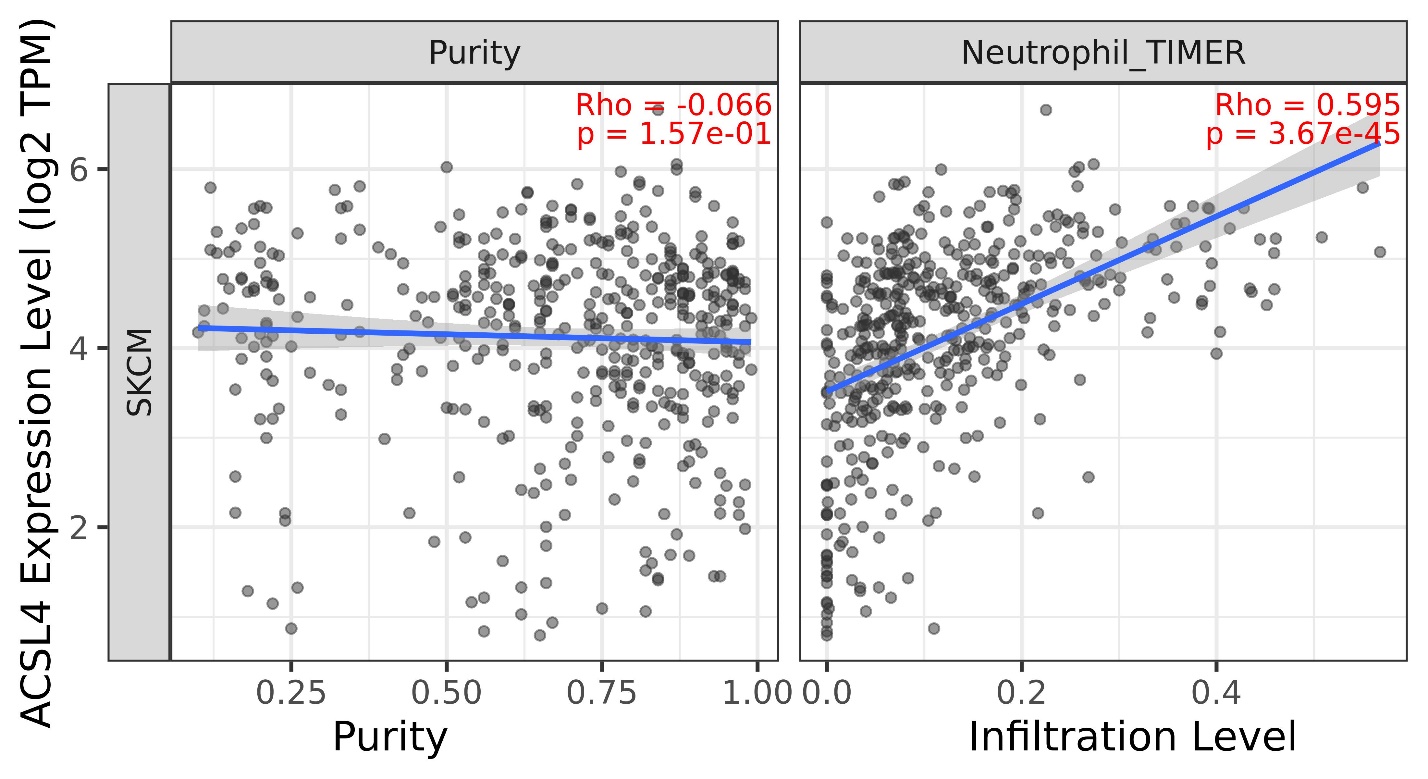


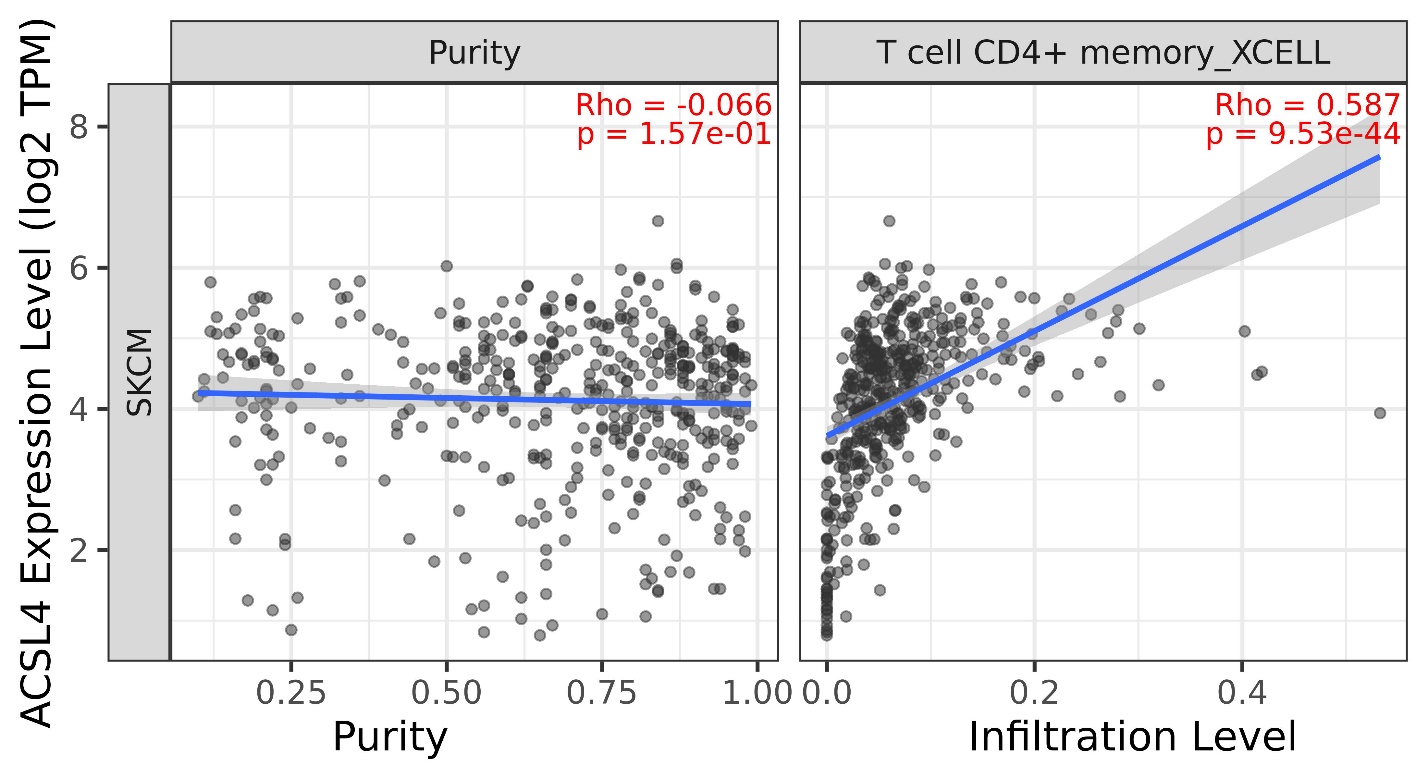


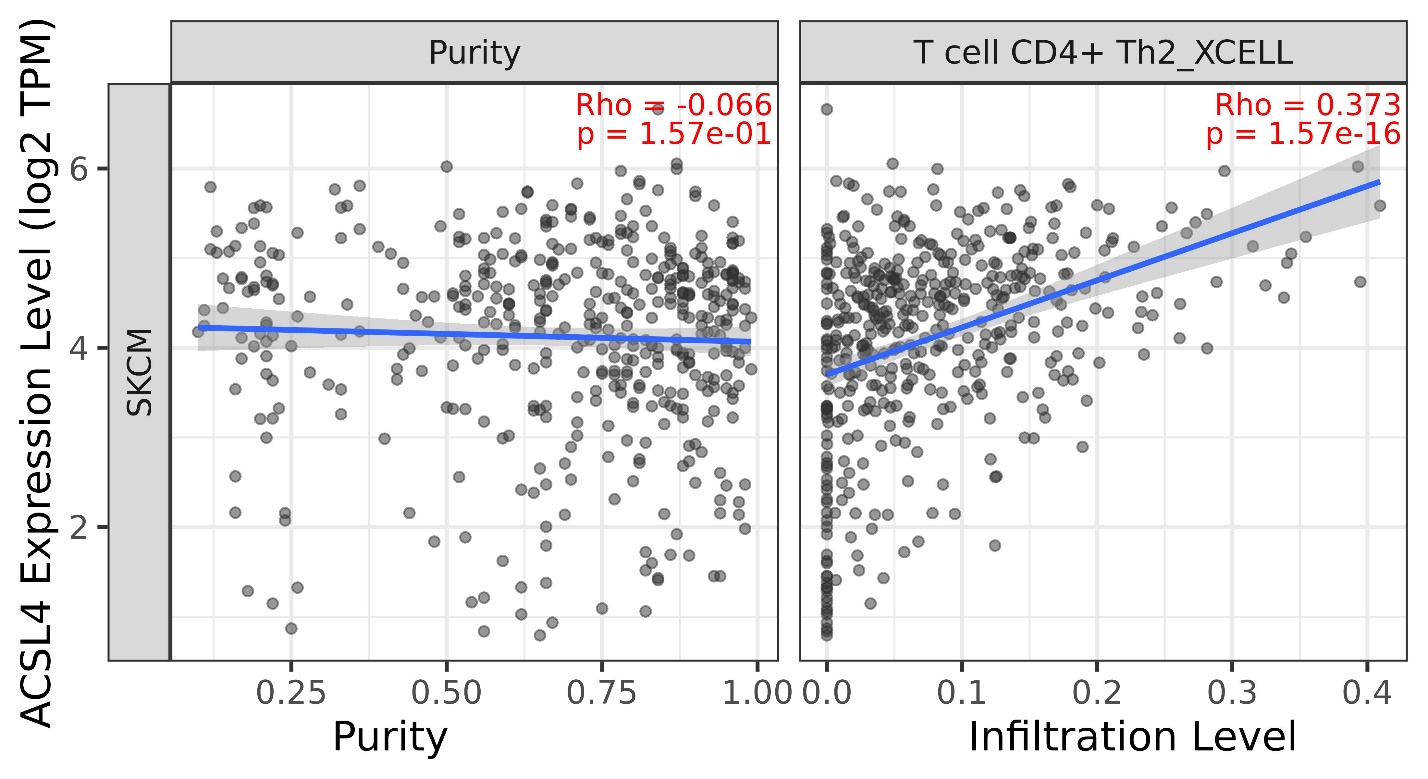


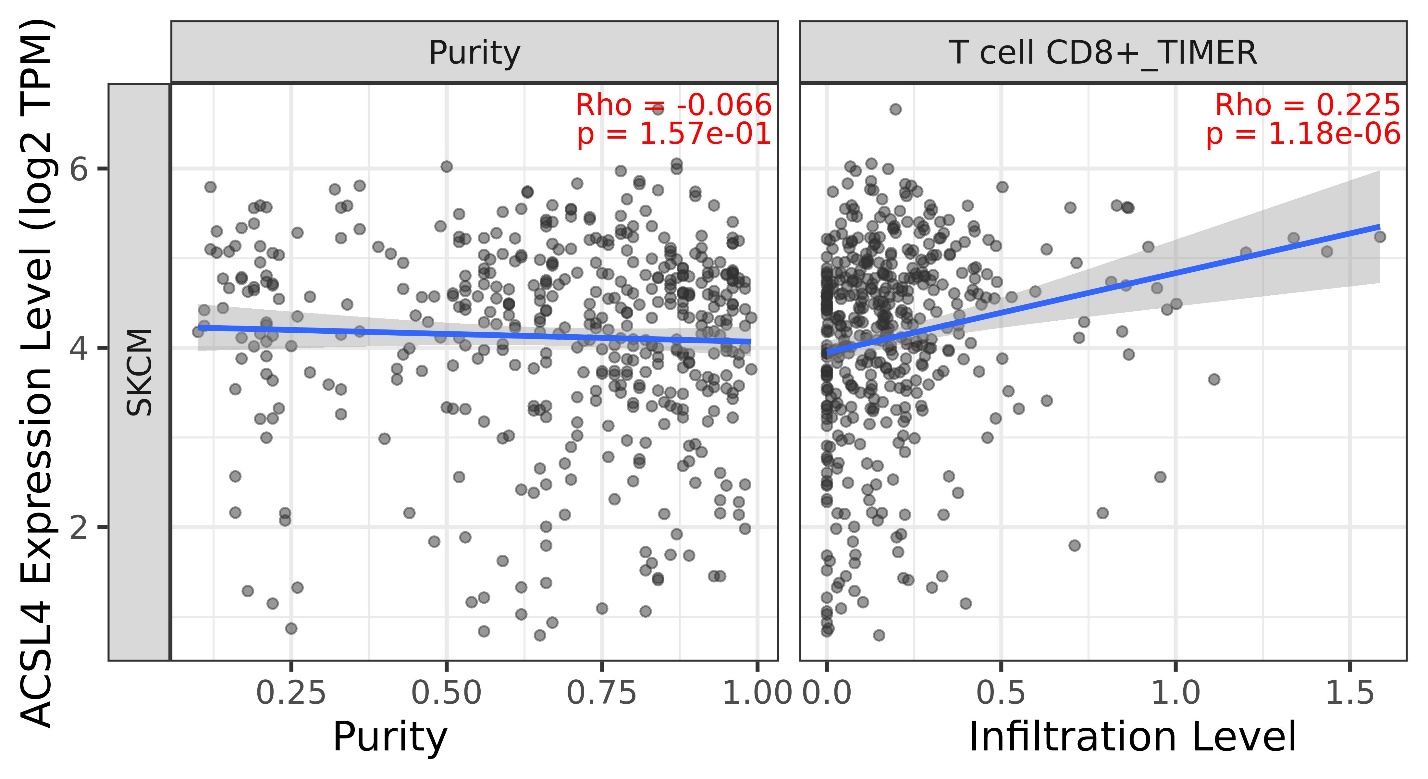


**Supplementary Figure S6. Scatter plots describing ACSL4 expression and tumor immune-infiltrating cells (TIICs).** Purity-adjusted correlations between ACSL4 mRNA levels and TIMER-estimated infiltration scores are shown for key immune/stromal populations. Panels depict B cells, cancer-associated fibroblasts (CAFs), M1 macrophages, monocytes, neutrophils, CD4⁺ T-cell subsets (memory, Th2), and CD8⁺ T cells; points represent individual tumors, with the correlation coefficient and p-value annotated on each panel. Overall, higher ACSL4 levels are associated with greater TIIC abundance.

| **Variable** | **Definition** | **Values** |
| --- | --- | --- |
| Loc_p | Localization of the diagnosed primary melanoma | 0 = trunk  1 = lower limbs  2 = upper limbs  3 = head and neck region  4 = acral region  5 = mucosal surface  6 = uvea |
| Loc_m | Localization of the diagnosed metastasis | 0 = lymph node  1 = subcutaneous tissue  2 = lungs  3 = liver  4 = central nervous system  5 = bone  6 = peritoneum  7 = ovary  8 = parotid  9 = small intestine  10 = large intestine  11 = spleen  12 = gallbladder  13 = kidney  14 = adrenal  15 = satellite  16 = subcutaneous  17 = muscle  18 = thyroid  19 = cartilage  20 = heart  21 = thorax  22 = gastric  23 = pelvis  24 = intestines  25 = breast  26 = pleura  27 = scrotum  28 = pancreas  29 = mesentery  30 = sinus  31 = choroid, uvea  32 = esophagus |
| Type | Subtype of malignant melanoma | Takes values 0, 1 or 2 depending on the subtype of melanoma  0 = superficial spreading melanoma (SSM)  1 = nodular melanoma (NM)  2 = acral lentininous melanoma (ALM) |
| T | Thickness of melanoma | 1 = Thickness < 1mm  2 = Thickness between 1 and 2 mm  3 = Thickness between 2 and 4 mm  4 = Thickness > 4 mm |
| U | Ulceration | Indicator variable:  1 in case of ulceration  0 otherwise |
| Clark | Depth stage of melanoma as it grows in the skin | 1 = Confined to the epidermis (the outer layer of the skin)  2 = Has invaded the papillary dermis (the outermost layer of the dermis, the next layer of skin)  3 = Has invaded throughout the papillary dermis reaching the superficial layer of the reticular dermis, or filled up the whole dermal papilla  4 = Has invaded this next deeper layer, the reticular dermis  5 = Has invaded the fat under the dermis |
| Breslow | Measure of depth of melanoma from the surface of skin to the deepest point of the tumour | This is a continuous variable that measures depth in mm |
| Regression | Decrease in the size of a tumour or in the extent of cancer in the body | 0 = No  1 = Yes |
| ACSL4 | Level of ACSL4 | This is a continuous variable that measures the level of ACSL4 |

**Supplementary Table S1. Explanatory variables.** Overview of covariates used in the melanoma analyses: age (years), sex (male/female), primary tumor site (trunk, lower limb, upper limb, head/neck, acral), melanoma subtype (SSM, NM, ALM), Breslow thickness (mm) and T category (T1-T4), ulceration (yes/no), Clark level (I-V), regression (yes/no), BRAF status (mutated/not), number of metastatic sites, and ACSL4 expression (continuous). Binary variables are coded 1 = yes, 0 = no. Covariate groupings (basic, genetic, histopathologic) are described in Section 2.2.

| **Variable** | **Type** | **Description** |
| --- | --- | --- |
| age | Numeric | Age in years |
| sex | Categorical | 1 = male  2 = female |
| loc_p_t | Categorical | 1 = primary melanoma diagnosed in trunk  0 = otherwise |
| loc_p_ll | Categorical | 1 = primary melanoma diagnosed in lower limbs  0 = otherwise |
| loc_p_ul | Categorical | 1 = primary melanoma diagnosed in upper limbs  0 = otherwise |
| loc_p_hn | Categorical | 1 = primary melanoma diagnosed in head and neck region  0 = otherwise |
| loc_p_a | Categorical | 1 = primary melanoma diagnosed in acral region  0 = otherwise |
| Loc_m_cnt | Numeric | Count of areas where metastasis has been diagnosed |
| Type0 | Categorical | 1 = SSM  0 = other type melanoma |
| Type1 | Categorical | 1 = NM  0 = other type melanoma |
| Type2 | Categorical | 1 = ALM  0 = other type melanoma |
| T | Categorical | 1 = Thickness of melanoma < 1mm  2 = Thickness between 1 and 2 mm  3 = Thickness between 2 and 4 mm  4 = Thickness > 4 mm |
| U | Categorical | 1 = Ulceration  0 = Otherwise |
| Clark | Categorical | Depth stage of melanoma  1 = Confined to the epidermis  2 = Has invaded the papillary dermis  3 = Has invaded throughout the papillary dermis and is touching on the next, deeper layer of the dermis  4 = Has invaded this next deeper layer, the reticular dermis  5 = Has invaded the fat under the dermis |
| Breslow | Continuous | Depth of melanoma in mm |
| Regression | Categorical | 1 = Decrease in the size of a tumor or in the extent of cancer in the body  0 = Otherwise |
| BRAF status | Categorical | 1 = BRAF mutated  2 = Otherwise |
| ACSL4 | Continuous | Level of ACSL4 |

**Supplementary Table S2. Final list of explanatory variables.** Final covariate set used in survival analyses. The table reports model coefficients (β), hazard ratios (HR, 95% CI),
and p-values for: age; sex (reference: male); primary tumor site indicators (trunk, lower limb, upper limb, head/neck, acral); number of metastatic sites; melanoma subtype indicators (SSM, NM, ALM); thickness category (T1-T4); ulceration; Clark level; Breslow thickness (mm); regression; BRAF status; and ACSL4 expression (continuous). HR>1 indicates increased hazard; HR<1 indicates decreased hazard. Coding and indicator construction are detailed in Supplementary Table 1 and the Methods section.

| **Variable** | **beta** | **HR (95% CI for HR)** | **p-value** |
| --- | --- | --- | --- |
| age | 0.0290 | 1.029 (1.00-1.10) | **0.047** |
| sex (reference: male) | -0.0180 | 0.835 (0.46-1.50) | 0.550 |
| loc_p_t | -0.0860 | 0.918 (0.52-1.60) | 0.770 |
| loc_p_ll | -0.1600 | 0.852 (0.38-1.90) | 0.690 |
| loc_p_ul | 0.1100 | 1.116 (0.56-2.30) | 0.750 |
| loc_p_hn | 0.2100 | 1.234 (0.55-2.80) | 0.600 |
| loc_p_a | 1.6000 | 4.953 (0.65-37.00) | 0.120 |
| Loc_m_cnt | -0.0034 | 0.997 (0.85-1.20) | 0.970 |
| Type0 | -0.1600 | 0.852 (0.48-1.50) | 0.580 |
| Type1 | 0.3400 | 1.405 (0.79-2.50) | 0.240 |
| Type2 | 1.6000 | 4.953 (1.20-23.00) | **0.030** |
| T | 0.4100 | 1.507 (1.10-2.10) | **0.010** |
| U | 0.4000 | 1.492 (0.83-2.70) | 0.190 |
| Clark | 0.3600 | 1.433 (1.00-2.00) | **0.043** |
| Breslow | 0.0730 | 1.076 (1.00-1.10) | **0.007** |
| Regres | -0.2300 | 0.795 (0.44-1.40) | 0.440 |
| BRAF status | -1.2000 | 0.301 (0.11-0.91) | **0.033** |
| ACSL4 | -13.0000 | 2.3e-06 (1.1e-09-0.011) | **0.002** |

**Supplementary Table S3. Univariate analysis for disease-free survival (DFS).**
Univariate Cox proportional hazards models for DFS in the melanoma cohort. For each covariate, the table reports the coefficient (β), hazard ratio (HR) with 95% CI, and p-value. HR>1 indicates higher hazard (shorter DFS); HR<1 indicates lower hazard (longer DFS). Variable definitions/coding follow Supplementary Table 1; p-value interpretation and multiple-testing control are described in Methods.

| **Variable** | **beta** | **HR (95% CI for HR)** | **p-value** |
| --- | --- | --- | --- |
| age | -0.0120 | 0.988 (0.95-1.00) | 0.490 |
| sex | 0.7500 | 2.117 (0.98-4.50) | 0.056 |
| loc_p_t | 0.0080 | 1.008 (0.47-2.20) | 0.980 |
| loc_p_ll | -1.1000 | 0.333 (0.081-1.40) | 0.140 |
| loc_p_ul | 0.4800 | 1.616 (0.64-4.00) | 0.310 |
| loc_p_hn | 0.3400 | 1.405 (0.48-4.10) | 0.540 |
| loc_p_a | 2.1000 | 8.166 (1.00-69.00) | 0.050 |
| Loc_m_cnt | 0.3600 | 1.433 (1.20-1.70) | **2.2E-4** |
| Type0 | -0.6400 | 0.527 (0.25-1.10) | 0.085 |
| Type1 | 0.6000 | 1.822 (0.85-3.90) | 0.130 |
| Type2 | 0.1400 | 1.150 (0.15-8.50) | 0.890 |
| T | 0.1900 | 1.209 (0.84-1.70) | 0.320 |
| U | 0.4500 | 1.568 (0.68-3.60) | 0.290 |
| Clark | 0.3200 | 1.377 (0.89-2.10) | 0.150 |
| Breslow | 0.0290 | 1.029 (0.94-1.10) | 0.510 |
| Regres | -0.2000 | 0.819 (0.37-1.80) | 0.620 |
| BRAF status | -0.6800 | 0.507 (0.12-2.20) | 0.360 |
| ACSL4 | 0.0790 | 1.082 (2.5e-05-4700) | 0.990 |

**Supplementary Table S4. Univariate analysis for metastasis-free survival (MFS).**
Univariate Cox proportional hazards models for MFS in the Szeged melanoma cohort. For each covariate, the table reports the coefficient (β), hazard ratio (HR, 95% CI), and p-value. HR>1 indicates higher hazard (shorter MFS); HR<1 indicates lower hazard (longer MFS). Variable definitions/coding follow Supplementary Table 1; modeling and p-value interpretation are described in Methods.

| **Variable** | **beta** | **HR (95% CI for HR)** | **p-value** |
| --- | --- | --- | --- |
| age | -0.0005 | 1.00 (0.97-1.00) | 0.980 |
| sex | -0.0067 | 0.99 (0.46-2.10) | 0.990 |
| loc_p_t | 0.0005 | 1.00 (0.48-2.10) | 1.000 |
| loc_p_ll | -1.1000 | 0.33 (0.08-1.40) | 0.130 |
| loc_p_ul | 0.5700 | 1.77 (0.75-4.10) | 0.200 |
| loc_p_hn | 0.2400 | 1.27 (0.44-3.70) | 0.670 |
| loc_p_a | 1.4000 | 4.06 (0.54-32.00) | 0.170 |
| Loc_m_cnt | 0.3100 | 1.36 (1.10-1.60) | **0.002** |
| Type0 | -0.2400 | 0.79 (0.38-1.60) | 0.520 |
| Type1 | 0.2200 | 1.25 (0.61-2.50) | 0.550 |
| Type2 | 0.0180 | 1.02 (0.14-7.50) | 0.990 |
| T | 0.3200 | 1.38 (0.95-2.00) | 0.088 |
| U | 0.2200 | 1.25 (0.58-2.70) | 0.570 |
| Clark | 0.3900 | 1.48 (0.97-2.30) | 0.069 |
| Breslow | 0.0840 | 1.09 (1.00-1.20) | **0.022** |
| Regres | -0.2700 | 0.76 (0.35-1.60) | 0.490 |
| BRAF status | -0.7800 | 0.46 (0.14-1.50) | 0.210 |
| ACSL4 | -7.0000 | 0.001 (1.8e-07-4.80) | 0.110 |

**Supplementary Table S5. Univariate analysis for overall survival (OS).** Univariate Cox proportional hazards models analyzing OS in the Szeged melanoma cohort. For each covariate, the table includes the coefficient (β), hazard ratio (HR with 95% CI), and p-value. An HR greater than 1 suggests increased hazard (shorter OS), while an HR less than 1 indicates decreased hazard (longer OS). Variable definitions and coding details are in Supplementary Table 1. Modeling assumptions and multiple-testing considerations are explained in the Methods section.
